# Supplementary material for: Enzobiotics—A Novel Therapy for the Elimination of Uremic Toxins in Patients with CKD (EETOX Study): A Multicenter Double-Blind Randomized Controlled Trial
Source: Nutrients. 2022 Sep 15;14(18):3804. doi: 10.3390/nu14183804 (PMC9503043; doi:10.3390/nu14183804)
Supplement: Supplementary file 1 [file nutrients-14-03804-s001.zip › Supplement S1. Nutrients.pdf]

Supplement – 1

QUANTITATION OF INDOXYL SULFHATE AND p-CRESOL IN CKD PATIENTS

Measurement System of PCS and IS

Recovery of Derivatised P-cresol and Indoxyl sulfate from serum was determined by comparing peak areas of extracted LQC, MQC and HQC samples with peak areas determined from freshly prepared unextracted (aqueous) samples prepared at similar concentrations in mobile phase.

Recovery of Internal standard (Obeticholic acid and Itraconazole) from serum was determined by comparing the peak areas of blank matrix samples spiked with IS (extracted samples) using this method with peak areas determined from freshly prepared IS (unextracted samples) in mobile phase at the concentration level required for the method.

Data Interpretation:

Mean overall % recovery was 85.36 % for P-Cresol and 93.65 % for Indoxyl sulfate

Mean Overall % recovery was 80.55% for Obeticholic acid (IS for Indoxyl sulfate) and 86.05% for Itraconazole.

Intra-Day- Derivatized P-Cresol

Intra-day precision (% CV) for P-Cresol LOQC samples ranged from 2.29% to 9.30% across the three precision and accuracy batches. Intra-day precision (% CV) for P-Cresol in LQC, MMQC, MQC and HQC samples ranged from 0.95% to 12.51%.

Intra-day accuracy (% nominal) for P-Cresol in LOQC samples ranged from 90.45% to 97.48% across the three precision and accuracy batches. Intra-day accuracy (% nominal) for P-Cresol in LQC, MMQC, MQC and HQC samples ranged from 88.07% to 100.28%.

Data Interpretation:

Intra-day precision and accuracy values were within established acceptance limits.

Intra-Day - <u>Indoxyl Sulfate</u>

Intra-day precision (% CV) for Indoxyl Sulfate LOQC samples ranged from 5.36% to 15.36% across the three precision and accuracy batches. Intra-day precision (% CV) for Indoxyl sulfate in LQC, MMQC, MQC and HQC samples ranged from 1.25% to 14.25%.

Intra-day accuracy (% nominal) for Indoxyl Sulfate in LOQC samples ranged from 90.45% to 97.48% across the three precision and accuracy batches. Intra-day accuracy (% nominal) for Indoxyl-sulfate in LQC, MMQC, MQC and HQC samples ranged from 92.72% to 106.52%.

Data Interpretation:

Intra-day precision and accuracy values were within established acceptance limits.

Sensitivity(LOQ)

The Sensitivity was assessed by considering the LOQ concentration. LOQ is spiked in to 6 replicates as QCs

Data Interpretation:

The mean derivatized p-Cresol accuracy and %CV were 98.34% and 5.40% respectively.

The mean Indoxyl sulfate accuracy and %CV were 98.34% and 5.40% respectively.

Limit of Detection (LOD)

Limit of detection was performed using biological matrix (serum) by decreasing the concentration of the analyte (i.e., p-Cresol and Indoxyl sulfate) by 2, 4, 8 ,16,32 times at LOQ and injected.

Data Interpretation:

Limit of detection of concentration for indoxyl sulfate was observed for 3.215 ng/ml

Limit of detection of concentration for p-Cresol was observed for 0.048 ng/ml

Internal Standard selection

Although the Internal standard selected for p-cresol and Indoxyl sulfate does not closely resemble like their analogues, the use of Itraconazole and Obeticholic acid can be justified as the Precision and Accuracy batches passed within the guidelines accepted criteria. If any interference altered the behaviour of analytes (p-cresol and Indoxyl sulfate) caused by these Internal standards might have seen in the result of Precision and Accuracy batches. But that scenario doesn't happened and the results are good.

This shows that the bioanalytical method employed using these internal standards proved to be rugged and satisfactory.

Mass spectrometry Conditions:
Make: AB Sciex 4000 QTRAP
Below table represents the mass parameters

Compound	m/z Q1 > Q3	Declustering potential	Entrance Potential	Collision Energy	Exit potential
Derivatised p-Cresol	342.3>171.1	95	10	36	15
Itracanazole (Internal standard)	705.1>392.3	83	10	33	16
Indoxyl Sulfate	211.9>79.9	60	10	28	15
Obeticholic acid (Internal standard)	419.2>419.2	75	10	35	16

S 1: Represents BLK+IS chromatogram of Indoxyl sulfate

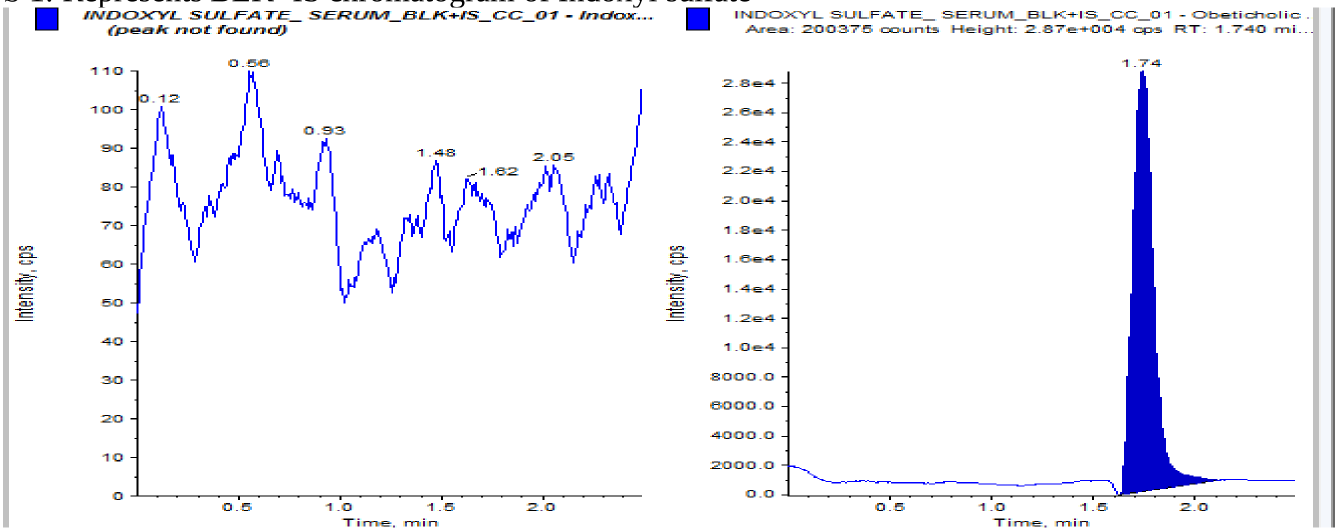

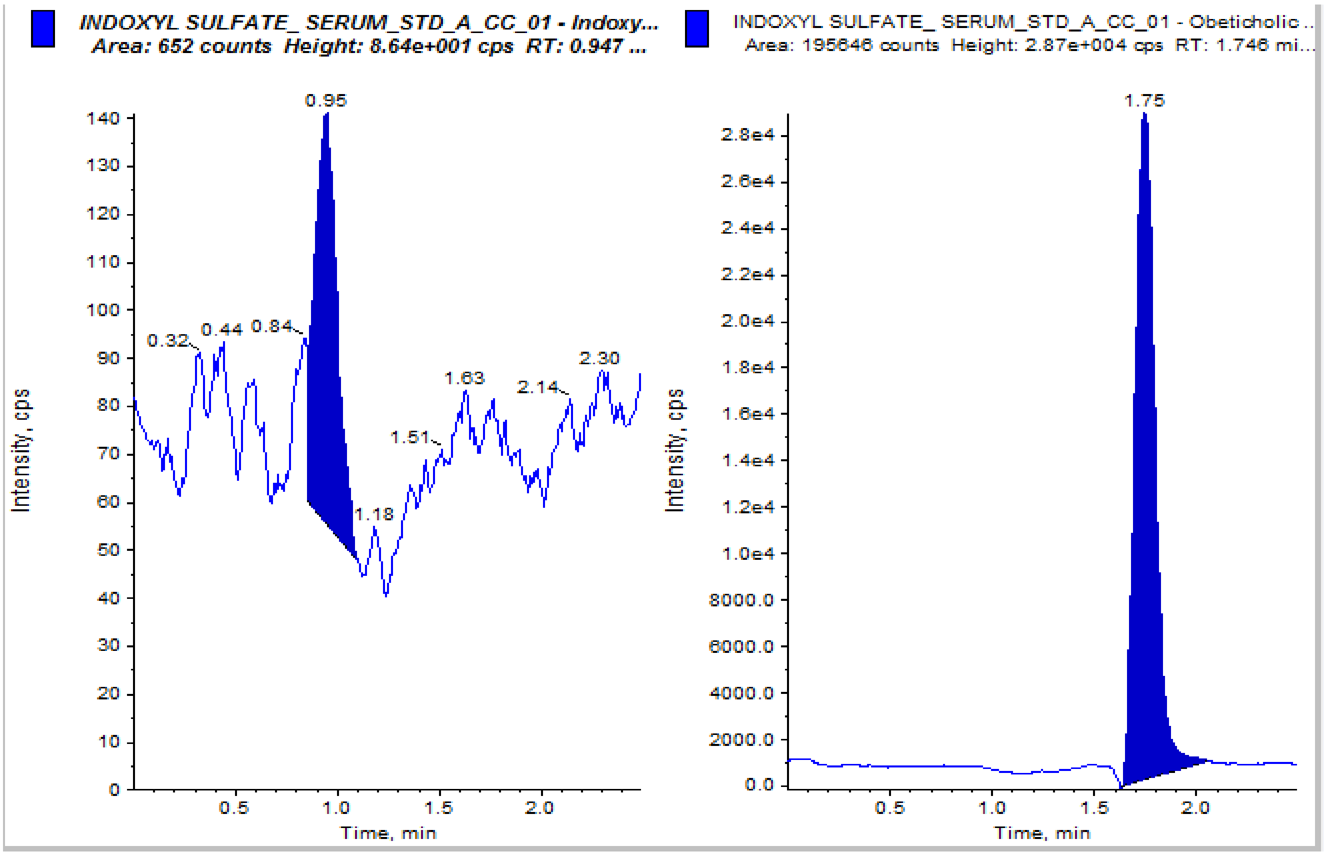

S 2: Represents STD A (105.790 ng/mL) chromatogram of Indoxyl sulfate

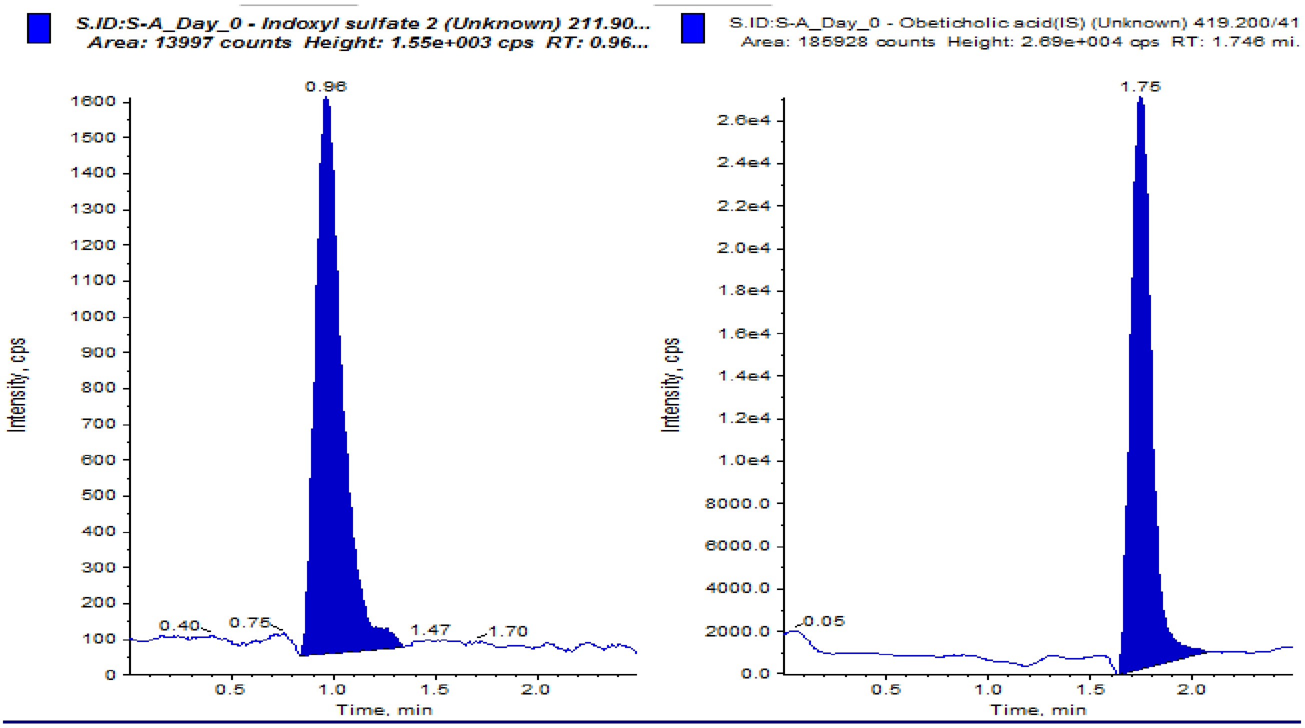

S 3: Represents Subject I.D : S-A at Day 0 (489.960 ng/mL)chromatogram of Indoxyl Sulfate

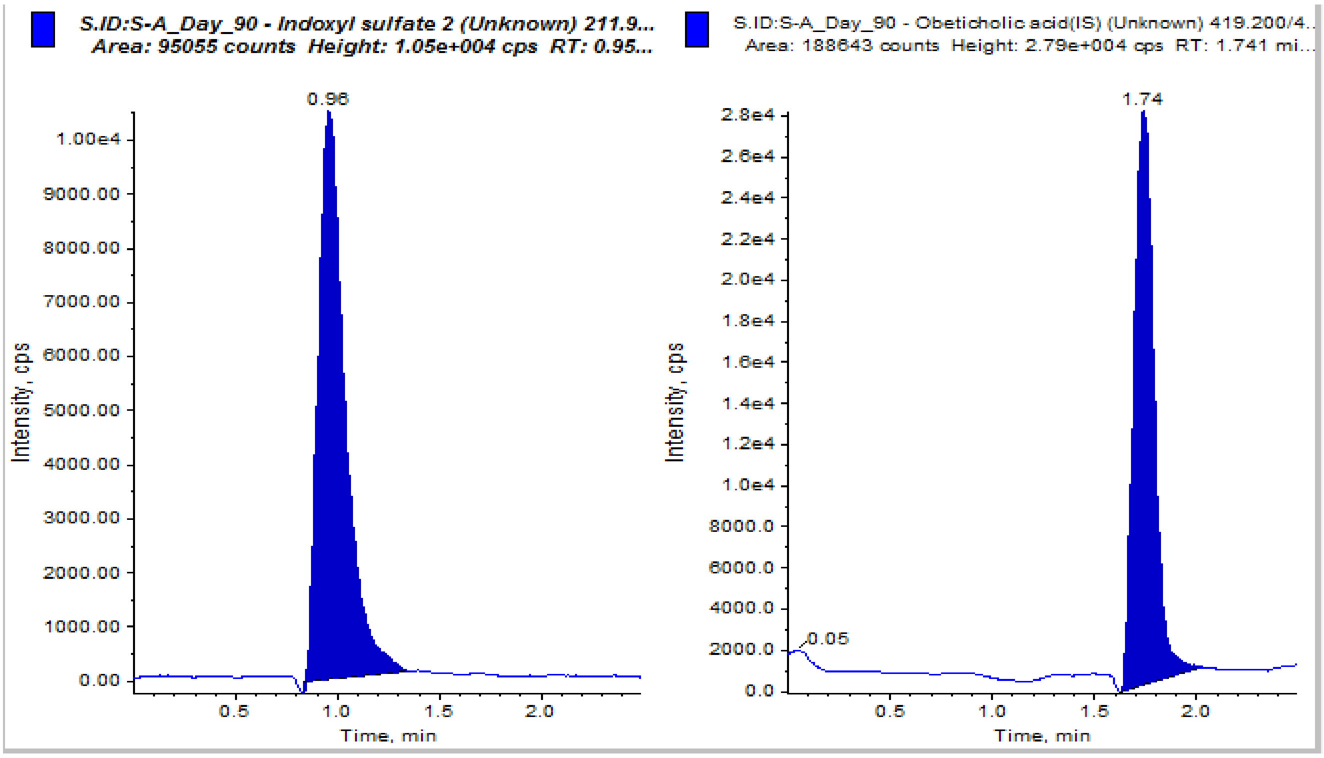

S 4: Represents Subject I.D :S-A at Day 90 (3962.569 ng/mL)chromatogram of Indoxyl Sulfate

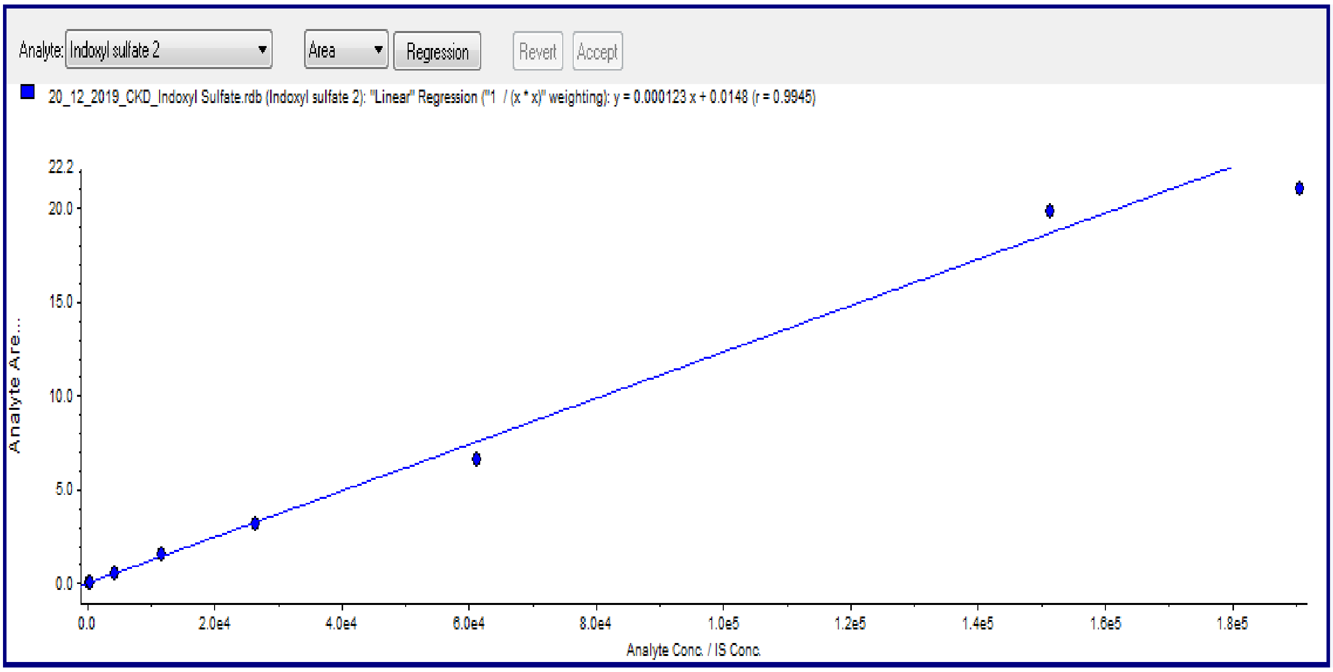

S 5: Represents the 8 point linearity curve of Indoxyl sulfate from 105.790 ng/mL to 95283.170 ng/mL .

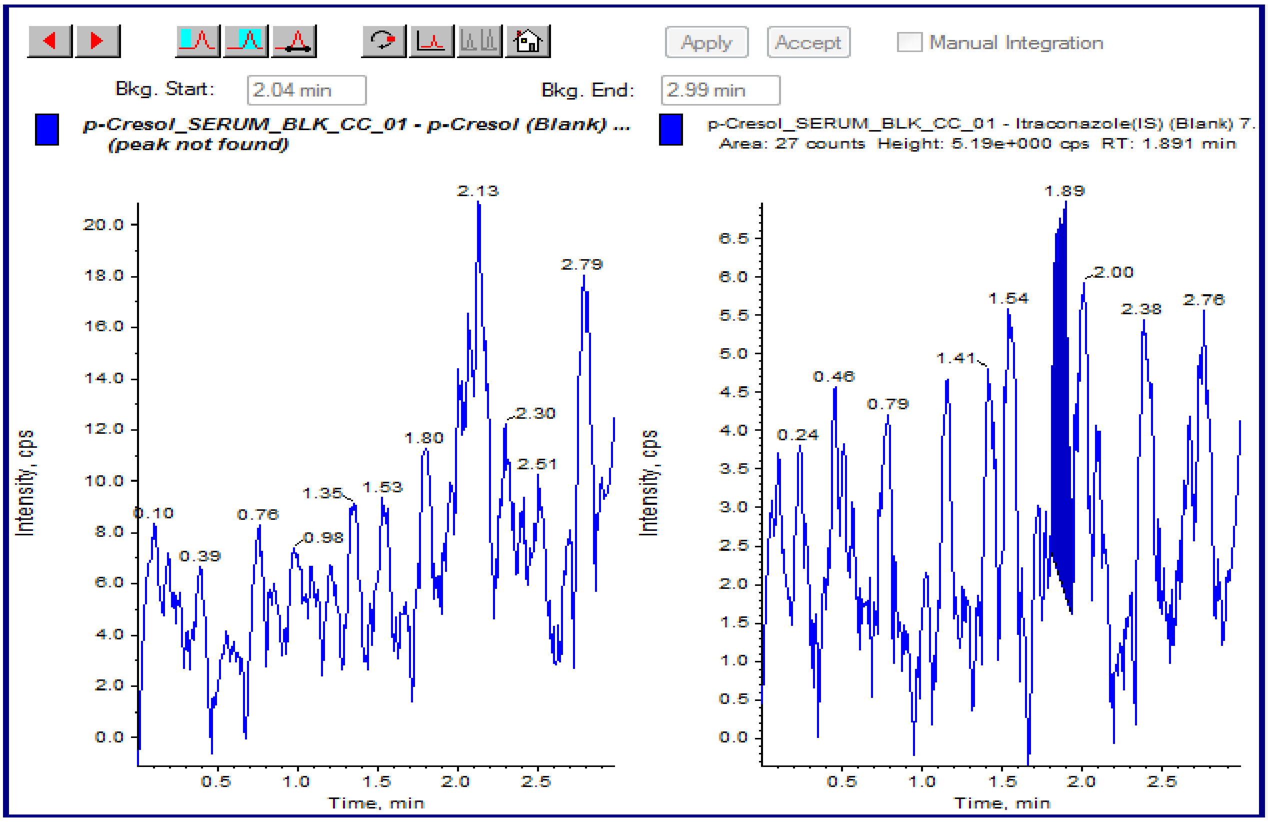

S 6: Represents BLK chromatogram of p-Cresol

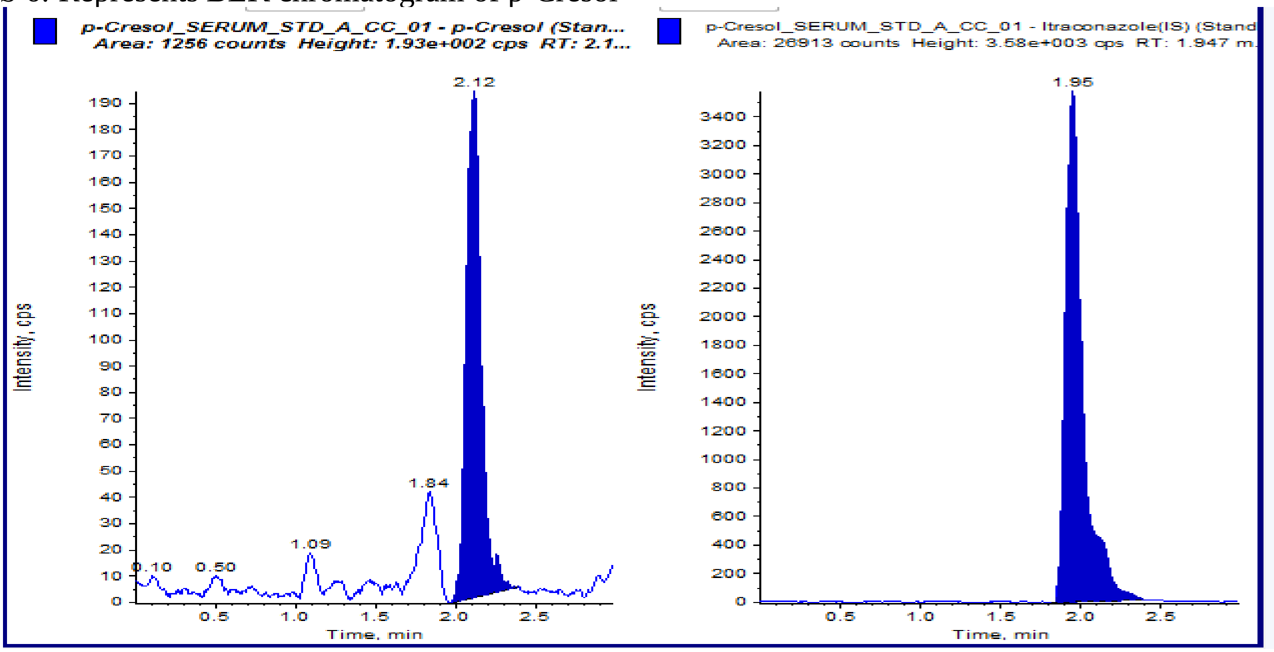

S 7: Represents STD A (0.096 ng/mL) chromatogram of p-Cresol

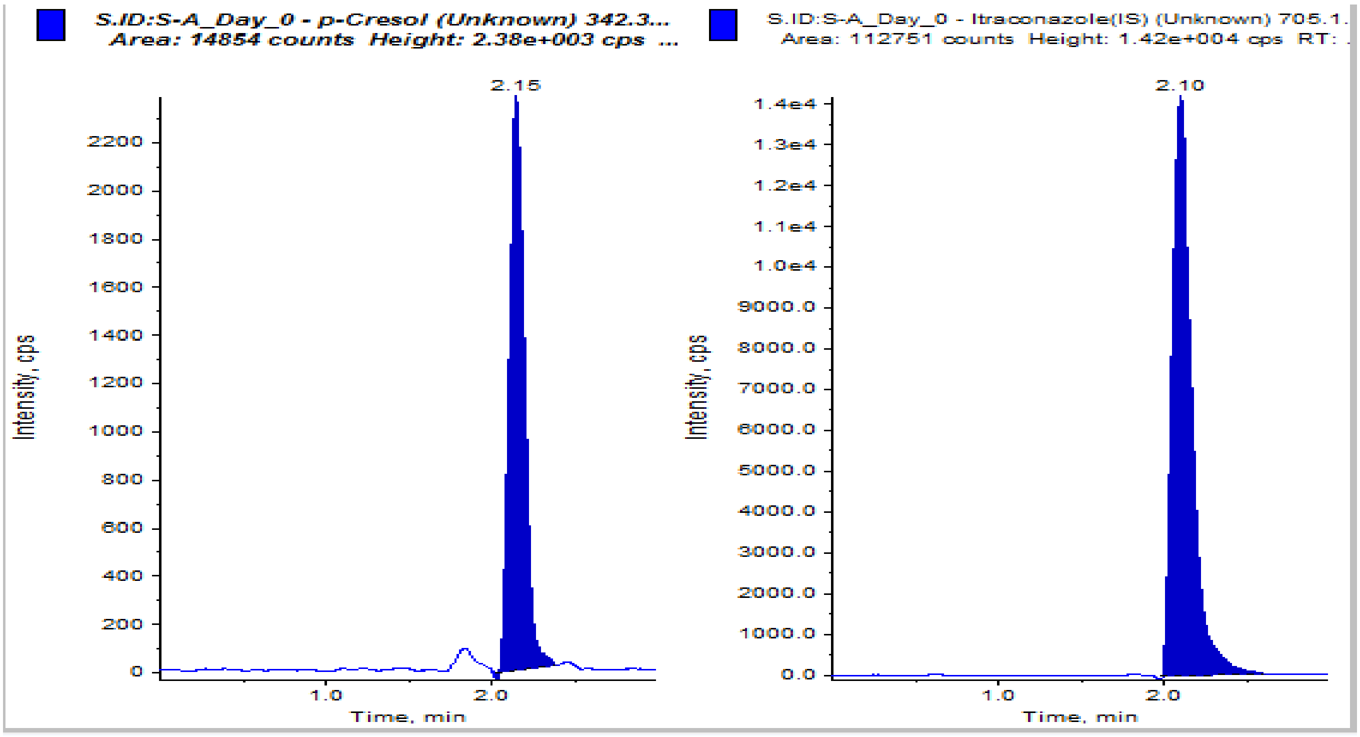

S 8:Represents Subject I.D :S-A at Day 0 (11.525 ng/mL)chromatogram .

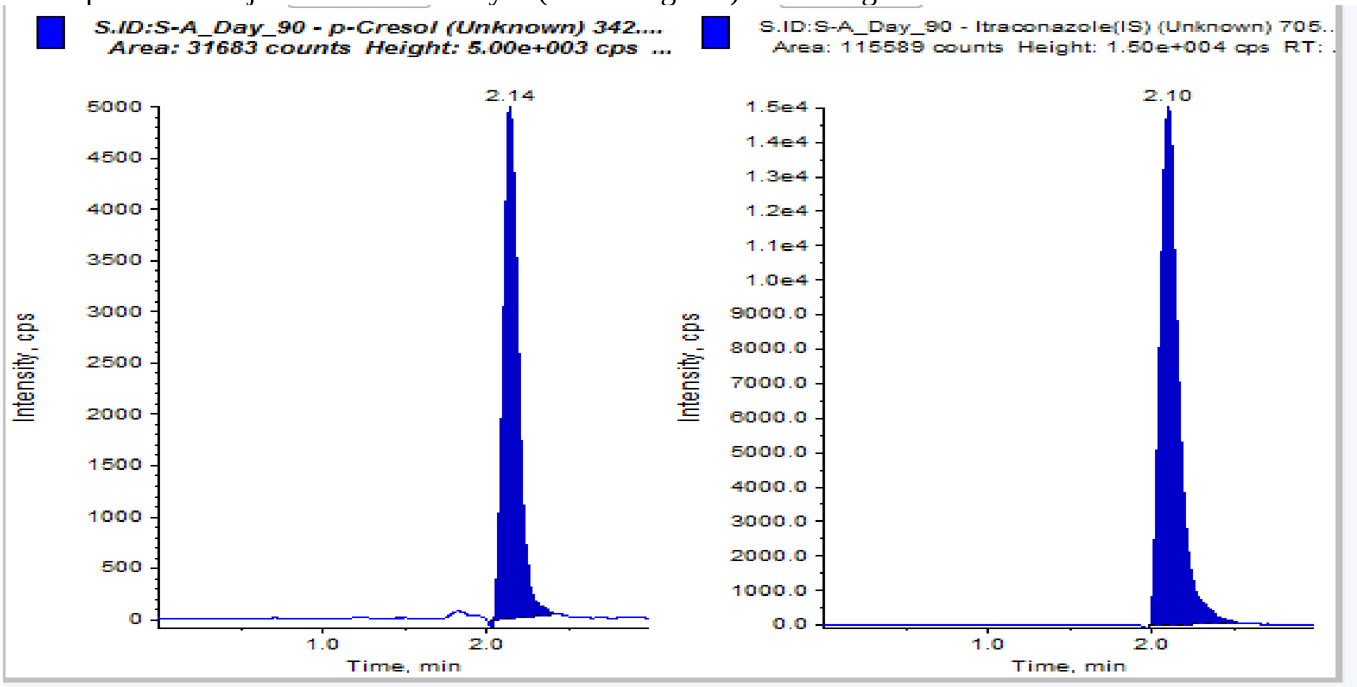

S 9:Represents Subject I.D :S-A at Day 90 (28.071 ng/mL)chromatogram .

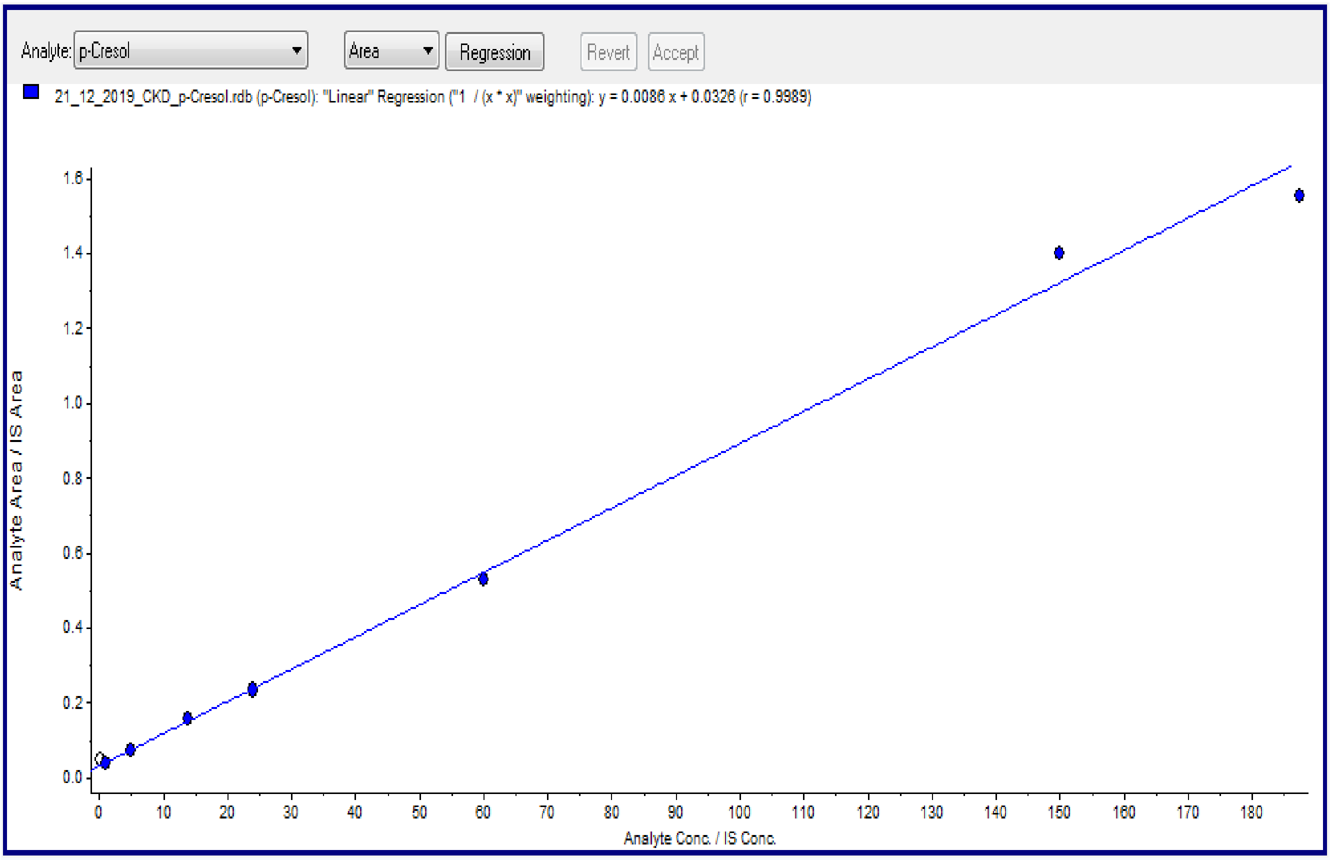

S 10: Represents the 8 point linearity curve of p-Cresol from 0.096 ng/mL to 187.488 ng/mL .
