# Supplementary material for: Enzobiotics—A Novel Therapy for the Elimination of Uremic Toxins in Patients with CKD (EETOX Study): A Multicenter Double-Blind Randomized Controlled Trial
Source: Nutrients. 2022 Sep 15;14(18):3804. doi: 10.3390/nu14183804 (PMC9503043; doi:10.3390/nu14183804)
Supplement: Supplementary file 1 [file nutrients-14-03804-s001.zip › Supplement S2. Nutrients.pdf]

Supplement -2

PCS and IS : Day-90/Day-0 Ratio

PLOS ONE 2014 guideline was followed for case Cohort study in comparing the improvement between Enzobiotics and placebo group.
The Changes in PCS and IS in Placebo and Enzobiotic between Day-90 and Day-0 represented as ratio of Day-90/ Day-0 values are given in Box Plot in below figure.

Figure S1: Changes in PCS and IS in Placebo and Enzobiotic groups on represented

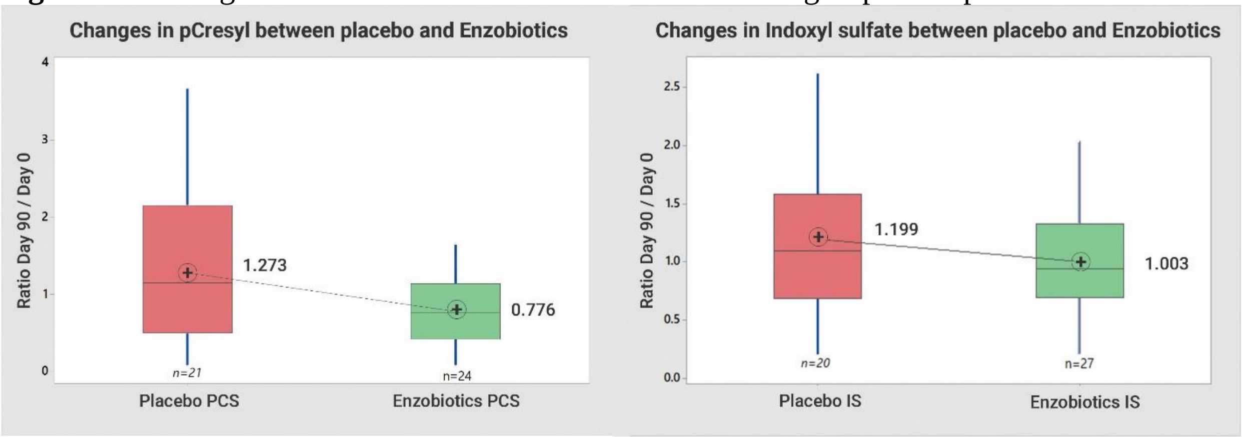

as ratio of Day-90/ Day-0 values

PCS:

The data is represented as box plot of Day-90/ Day-0 .PCS and IS ratio. The PCS increased by +27% (ratio of Day-90/ Day-0, n=21, SD + 0.93, CI 0.85 to 1.69,), in Placebo vs an improvement by - 23% reduction %(ratio of Day-90/ Day-0, n=24, SD +0.483, CI from 0.60 to 0.96). in Enzobiotics group (p=0.012). An improvement by1.64 times was noted. The homogeneity of patients in Enzobiotic group was more consistent (SD 0.424) compared to Placebo group (SD 0.928).

IS:

The ratio of (Day-90 / Day-0) IS in Placebo group was 1.199 an increase of 20% (1.20+- 0.71) compared to Enzobiotics group 1.003(1.003+-0.5) at 0.3% indicating stabilization of toxin levels. The Standard error for mean was 0.16 for Placebo and 0.10 for Enzobiotics

The Individual distribution of PCS atDay-0 and Day-90 for each group (Enzobiotics and Placebo) are given in Figure below

Figure S2 : Distribution of PCS for Placebo Day-**0 ,** Day-**90 &Enzobiotics** Day-**0 ,** Day-**90**

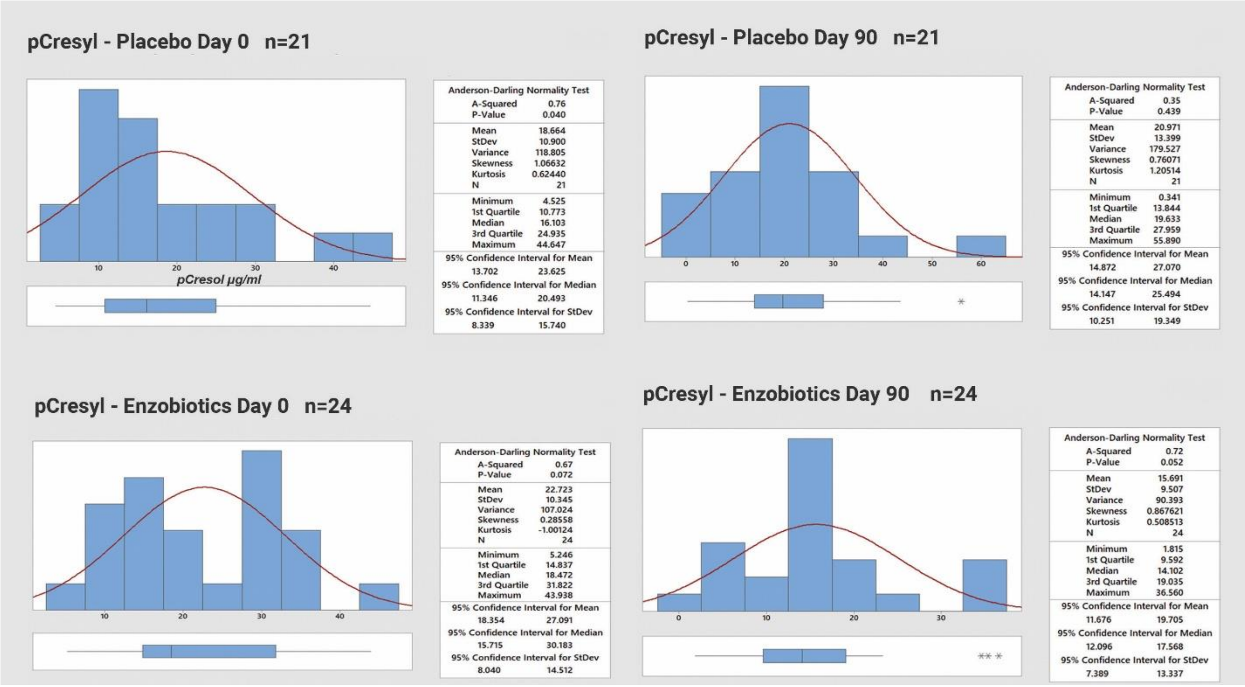

The propensity of randomness Day-0 at the time of recruitment between the group for efficient comparison was established with no significant difference in PCS (p= 0.270) between Placebo (18.7 ±10.9) and Enzobiotic groups at Day-0 (22.7 ±10.3). The variance was equal between the groups (F Test P= 0.803), at initial „ Day-0‟ and improvement after Day-90 between two groups was observed.

The absolute mean value of PCS increased by 12% from18.66 at Day-0 (n= 21 ,SD10.9 and CI:13.70 to 23.62)to20.97µgm/ml at Day-90 (n= 21, SD 13.4 CI : 14.87 to 27.07) for Placebo group and reduced by 31 % from 22.72 µg/ml at Day-0 (n=24 SD 10.34 CI ;18.35 to 27.09) to 15.69µg/mL (n=24 SD 9.51CI: 11.68 to19.70).

The Individual distribution of IS atDay-0 and Day-90 for each group (Enzobiotics and Placebo) are given in Figure below.

Figure S3: **Distribution of IS between Day-0 and Day-90 comparing Placebo and Enzobiotics group**

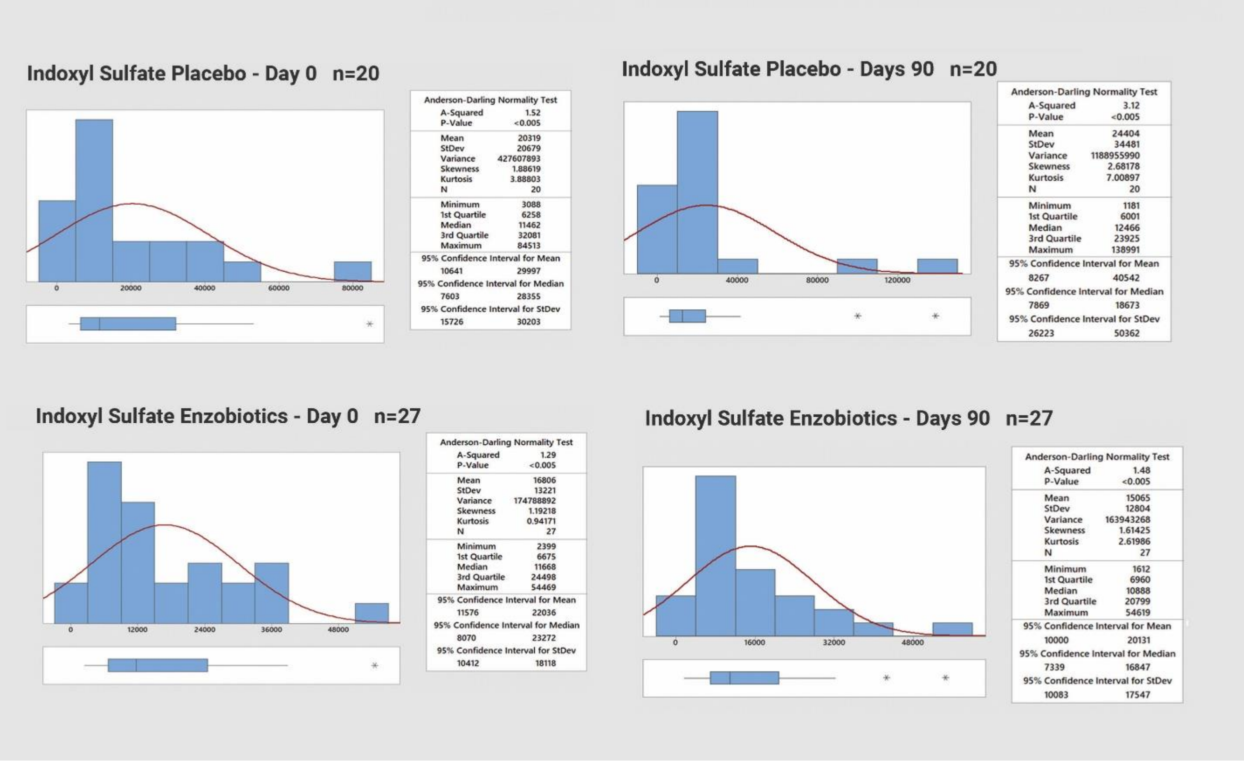

The absolute **median** value of IS (based on Lognormal model distribution) increased by 8.8% from 11462ng/ml (Day-0) (n=20 SD 20679 CI median :7603-28355) to 12466ng/ml (Day-90) (n=20 SD 34481ng/ml CI median :7869-18673)for Placebo group and reduced by 6.7% from 11668 (Day-0) (n=27SD 13221CI median from 8070 to 23272) to 10888 ng/mL (n=27 SD 12804CI median : from 7339 to 16847) for Enzobiotic group, noticing the consistency in standard deviation
