# Supplementary material for: Enzobiotics—A Novel Therapy for the Elimination of Uremic Toxins in Patients with CKD (EETOX Study): A Multicenter Double-Blind Randomized Controlled Trial
Source: Nutrients. 2022 Sep 15;14(18):3804. doi: 10.3390/nu14183804 (PMC9503043; doi:10.3390/nu14183804)
Supplement: Supplementary file 1 [file nutrients-14-03804-s001.zip › Supplement S3. Nutrients.pdf]

**Supplement - 3**

**Diagnostic Prediction:**

STREGA / GRIPS guidelines has been followed to determine the relationship between variables and the diagnostic prediction of Toxin, identifying the predictors from the multivariate analysis.

The dimensional reduction of 34 study variables for prediction was carried out using principle component analysis measuring the Eigen value and loading value. Eigen value is contribution of each factor to total variation out of 34 variables.Loading plot reveals the contribution of each characteristic influences the principle component, Ref table below

**Table S1**: Principle Components for Loading Plot

| Variable | PC1 | PC2 | PC3 | PC4 | Variable | PC1 | PC2 | PC3 | PC4 |
|---|---|---|---|---|---|---|---|---|---|
| HT | 0.097 | -0.112 | 0.016 | 0.173 | Hg (hemoglobin) | 0.261 | 0.073 | -0.258 | 0.123 |
| WT | 0.184 | 0.212 | 0.261 | -0.013 | HMT | 0.280 | 0.095 | -0.218 | 0.116 |
| BMI | 0.133 | 0.268 | 0.253 | -0.131 | Albumin | 0.212 | -0.131 | -0.300 | 0.112 |
| Temp | 0.125 | 0.063 | 0.001 | -0.178 | Na | 0.158 | -0.164 | 0.050 | -0.141 |
| HR | -0.034 | -0.065 | -0.297 | -0.357 | K | 0.115 | 0.100 | 0.092 | 0.243 |
| PR | -0.032 | -0.075 | -0.300 | -0.355 | Urea | -0.279 | 0.194 | -0.109 | 0.149 |
| RR | 0.038 | -0.082 | 0.056 | -0.031 | U.A | -0.036 | 0.096 | 0.008 | 0.191 |
| Systolic | 0.026 | 0.215 | -0.135 | -0.022 | TP | 0.165 | 0.092 | -0.192 | 0.107 |
| Diastolic | 0.102 | 0.064 | -0.226 | -0.106 | Triglycerides | 0.189 | 0.207 | -0.161 | 0.048 |
| PC | -0.014 | 0.030 | 0.146 | -0.158 | TC | -0.005 | 0.416 | -0.111 | -0.189 |
| WBC | 0.134 | 0.156 | -0.008 | -0.096 | EGFR9MDRD | 0.278 | -0.060 | 0.132 | -0.098 |
| RBC | 0.239 | 0.125 | -0.255 | 0.059 | LDL | -0.018 | 0.403 | -0.108 | -0.193 |
| SGOT | 0.127 | 0.155 | 0.053 | 0.345 | HDL | -0.102 | 0.144 | 0.110 | -0.118 |
| SGPT | 0.163 | 0.066 | 0.054 | 0.337 | EGFR-CGF | 0.289 | -0.028 | 0.174 | -0.120 |
| Total Bilirubin | 0.136 | -0.077 | 0.174 | -0.060 | HBA1c | 0.039 | 0.293 | 0.179 | -0.122 |
| BUN | -0.279 | 0.194 | -0.110 | 0.149 | P | -0.232 | 0.176 | 0.046 | 0.146 |
| Creatinine | -0.306 | 0.104 | -0.114 | 0.065 | hsCRP | 0.045 | 0.181 | 0.249 | -0.121 |

**Legend : PC1-PC4** –Principle component. The Linear combination of the factors are shown as PC1-PC4.Highest negative contrast were taken as predictors.

**Figure S4:** Eigen Value Scree Plot and Eigen Vector Loading Plot

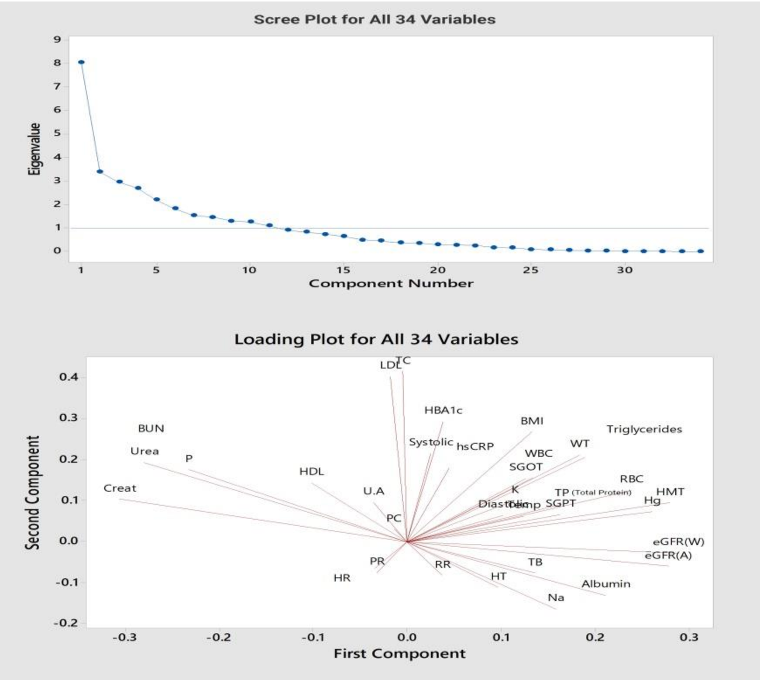


Eighty two percent (82%) of the structural variation, was explained by 11 principal variables out of 34 variables, Nine negative contrast variables (creatinine, BUN, urea, phosphorus, HDL, uric acid, heart rate, pulse rate and platelet count) were identified. The two positive contrast variables were eGFR, and RBC.

Multiple linear regression, identified the significant linear predictors for PCS at Day-0

<u>**Predicted Equation**</u>: PCS**(**Day-**0)** = - **5.97** **+ 0.0453* PC** **+ 2.987* U.A - 1.310 *Creatinine**

Multiple linear regression, identified the significant linear predictors for PCS at Day-90 -for Enzobiotics and Placebo groups.

Similarly for Day-90 PCSthe significant predictor were Platelet Count and initial PCS at Day-0

<u>**Predicted Equation**</u>

B – Placebo**PCS(**Day-**90)= 28.62-0.0542* PC + 0.283 *** PCS**(**Day-**0)**

A – Enzobiotic**PCS (**Day-**90)= 22.92-0.0542* PC + 0.283 *** PCS**(**Day-**0)**

The results show that when PC (platelet count) increases by 1 unit reduced PCS by 0.05 µg/ml and 3 µg/ml for every unit increase of Uric Acid. Enzobiotics reduces PCS by 20% from 28.62 in Placebo to 22.92 in Enzobiotics

The prediction of PCS at Day-90 is graphically represented in contour plot shown below.

**Figure S5 :** shows P Cresol Day-90 on PC and PCS base zero for Placebo group

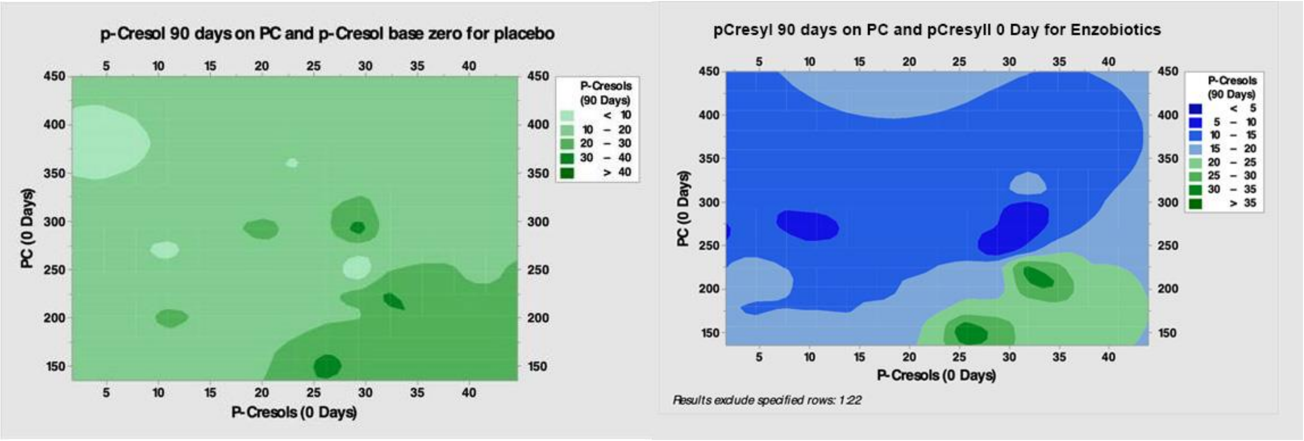


**Legend**: The figure shows relationship between Day-0 PCS (x-axis), Day-0 platelet count (y-axis) and colour coded image showing the Day-90 PCS for Placebogroup.

When PC decreases below 250, the PCS increases beyond 20µg/ml at Day-0. In Enzobiotics group, after Day-90, when platlet count was between 200 to 400 the PCS reduced to less than 15 µg/mL In Placebo group, after Day-90, with platlet count below 200 PCS was above 20µg/mL. This multiple linear regression approach determines the threshold level of urimic toxin.(20 µg/ml for PS and 20000 ng/ml for IS) as observed from the figures.

Further external data were taken in order to validate the association of creatinine with uric acid and platelet count with a larger sample (n=585**).** The results show that though creatinine level increased based on age, gender, and CKD stage, the creatinine level reduced by 0.3mg/dL for every million increase in platelet count. However the findings of the investigators need to be validated in a different setting and in a larger cohort as not much work has been carried out on prediction of uremic toxin using a combination of commonly available lab tests that is presented here

The three dimensional contour plot as nomogram on the changes in IS Level(ng/ml) in respect of diagnostic biomarkers based on the above equation determines the threshold level of IS at 20,000mg/ml shown in figure below. The initial IS level at Day-0 was also significant predictor after 90 days for every patient.Hence IS at Day-0 was predicted from creatinine alone whereas at 90 days , all thre uremic parameters are also determining the IS Day-90.

**Figure S6:** Contour plot Nomogram showing Threshold level of PCS and IS determining uremic parameters platelet count and serum Creatinine

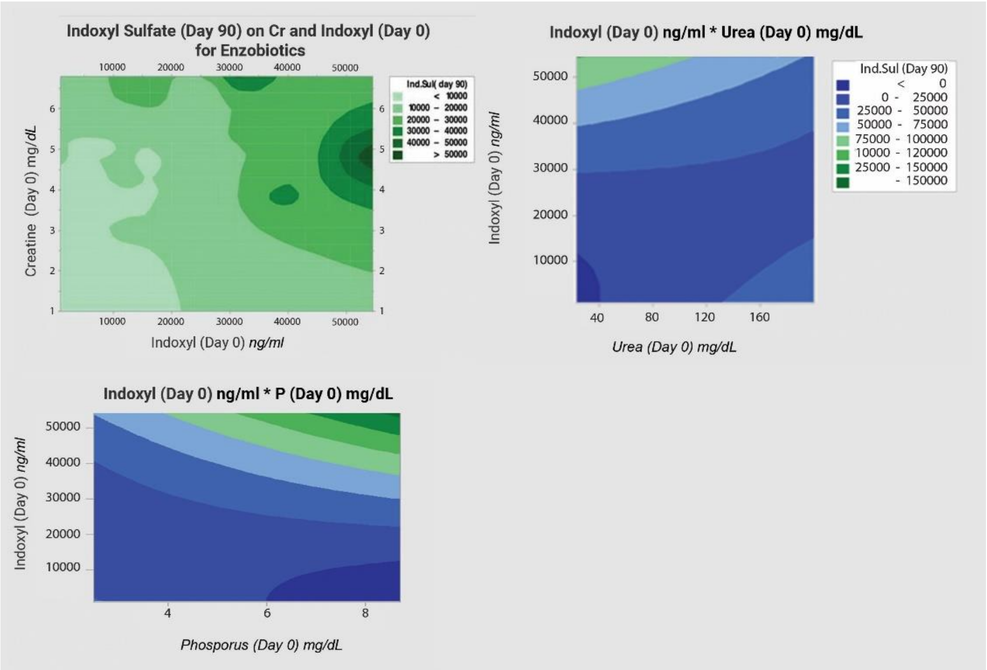


If the serum creatinine increases by 1 mg/dl, IS increases by 4689ng/dl; if serum urea increases by 1 mg/dl, IS reduces by 243ng/dl; and if serum phosphorus increases by 1 mg/dl, ISincreses by 3946ng/dl significantly.The threshold level of IS could be be vividly seen around 20,000mg/ml from the contour diagram for the changes in three uremic diagnostic predictors.
