# Supplementary material for: Enzobiotics—A Novel Therapy for the Elimination of Uremic Toxins in Patients with CKD (EETOX Study): A Multicenter Double-Blind Randomized Controlled Trial
Source: Nutrients. 2022 Sep 15;14(18):3804. doi: 10.3390/nu14183804 (PMC9503043; doi:10.3390/nu14183804)
Supplement: Supplementary file 1 [file nutrients-14-03804-s001.zip › Supplement S4. Nutrients.pdf]

Supplement 4

Table S2 : External data validation in N=585 Creatinine on Platelet Counts and Uric Acid according to age and Gender

Patient Code	Gender	Age	Request Date	Creatinine Value	GFR	CKD _Class	Uric Acid Value	Platelet Count Value
201907170015	Male	58	24-07-2019	4.39	14.8	5	1.2	175000
201811090071	Male	55	9/11/2018	3.43	19.9	4	1.4	155000
201804170048	Male	49	3/5/2018	10.23	5.8	5	1.5	218000
200907060071	Female	21	2/3/2018	0.64	124.7	1	1.5	261000
201906050004	Male	56	5/6/2019	0.61	145.6	1	1.5	241000
201810020102	Male	52	2/10/2018	1.3	61.7	2	2	159000
201909040117	Female	45	4/9/2019	0.39	189.2	1	2.2	601000
201810080031	Male	55	8/10/2018	2.89	24.3	4	2.5	248000
201809240066	Male	41	1/10/2018	7.98	8	5	2.6	180000
201801260053	Male	28	30-01-2018	4.81	15.5	4	2.6	251000
201912060035	Male	42	6/12/2019	11.19	5.4	5	2.7	365000
201809280041	Male	60	15-10-2018	5.7	10.9	5	2.7	152000
200612180129	Female	68	29-07-2019	1.8	29.8	4	2.7	256000
KGH-201512290069	Female	50	8/4/2019	0.58	117.1	1	2.7	215000
201807200038	Female	37	20-07-2018	0.53	138.2	1	2.7	399000
201902080090	Male	50	8/2/2019	6.29	10.1	5	2.8	157000
201809270127	Male	47	29-11-2019	2.77	26.3	4	2.8	155000
201803220004	Female	58	22-03-2018	6.81	6.6	5	2.9	237000
201802090039	Male	57	15-06-2019	3.07	22.5	4	2.9	181000
201802100050	Female	82	10/2/2018	1.29	42.1	3	3	233000
200105080089	Male	73	6/9/2018	1.39	53.3	3	3	291000
201902250031	Female	59	25-02-2019	0.92	66.5	2	3	371000
201402090004	Female	39	2/4/2019	0.8	85	2	3	221000
201808130043	Female	27	13-08-2018	0.52	150.6	1	3	414000
201812170005	Female	39	17-12-2018	0.44	169.5	1	3	15000
201801100052	Male	50	10/1/2018	0.78	112.2	1	3.1	397000
201801260053	Male	29	5/4/2019	5.5	13.2	5	3.2	222000
201809080086	Female	39	21-09-2018	3.74	14.3	5	3.2	165000
201905270023	Female	49	27-05-2019	0.47	149.9	1	3.2	280000
201903180059	Male	71	18-03-2019	5.57	10.8	5	3.3	245000
201812040032	Male	58	4/12/2018	4.59	14.1	5	3.3	211000
201901020039	Female	33	2/4/2019	2.11	28.7	4	3.3	161000
201901210039	Male	33	21-01-2019	0.78	122	1	3.3	331000
201812140048	Male	67	14-12-2018	0.6	143.1	1	3.3	121000
201805160006	Male	36	16-05-2018	8.96	7.2	5	3.4	209000
201802210043	Female	59	27-03-2018	4.15	11.7	5	3.4	208000
201411110063	Female	81	8/10/2019	1.72	30.3	3	3.4	246000

201906120017	Male	53	27-06-2019	5.22	12.4	5	3.5	343000
201902140055	Male	58	14-02-2019	4.78	13.4	5	3.5	240000
200212310099	Male	77	28-03-2019	3.57	17.8	4	3.5	162000
201311300035	Female	55	19-02-2018	1.14	52.7	3	3.5	286000
201802220023	Male	56	22-02-2018	1.29	61.3	2	3.5	179000
200711200031	Male	35	26-04-2018	9.64	6.6	5	3.6	317000
201803190034	Male	40	13-05-2019	9.01	7	5	3.6	328000
201806190108	Male	52	19-06-2018	7.56	8.1	5	3.6	180000
201806210063	Male	38	4/10/2018	6.85	9.7	5	3.6	181000
201812050114	Male	32	5/12/2018	5.32	13.4	5	3.6	229000
201802220055	Female	41	22-02-2018	0.6	117.3	1	3.6	245000
201903080047	Female	29	12/4/2019	5.64	9.5	5	3.7	220000
201908020136	Male	70	14-11-2019	6.24	9.5	5	3.7	169000
201811260076	Male	30	28-11-2018	7.1	9.7	5	3.7	461000
201708040092	Male	43	24-01-2018	6.07	10.8	5	3.7	210000
201109210124	Female	39	17-04-2019	4.62	11.2	5	3.7	341000
201806210027	Female	49	9/8/2018	0.59	115.3	1	3.7	418000
201904090034	Male	24	29-08-2019	5.75	13	5	3.8	268000
201902280108	Male	72	14-03-2019	3.92	16.2	4	3.8	268000
201905110038	Female	62	11/5/2019	1.38	41.2	3	3.8	387000
201803150047	Male	75	15-03-2018	1.48	49.3	3	3.8	225000
200908200041	Female	25	18-07-2019	1.3	53.1	3	3.8	197000
201812050075	Male	53	11/12/2018	6.48	9.6	5	3.9	174000
201802140106	Female	53	14-02-2018	4.44	11.1	5	3.9	225000
201401270069	Male	42	3/1/2019	5.41	12.4	5	3.9	159000
201704270046	Female	62	3/4/2018	0.91	66.7	2	3.9	289000
201909120049	Male	60	12/9/2019	1.17	67.7	2	3.9	188000
201812310110	Female	53	1/1/2019	0.67	98	1	3.9	204000
201907290063	Male	69	29-07-2019	0.71	117.1	1	3.9	434000
KGH-201502270153	Male	67	18-11-2019	4.11	15.5	4	4	367000
201805290054	Male	41	29-05-2018	2.1	37.2	3	4	254000
201908220032	Female	55	22-08-2019	0.55	122.2	1	4	320000
201405130026	Female	40	30-11-2019	0.51	142.2	1	4	380000
201807250025	Male	44	14-11-2018	7.33	8.7	5	4.1	183000
201910070040	Female	54	7/10/2019	5.34	8.9	5	4.1	258000
201812030078	Male	50	19-10-2019	6.92	9	5	4.1	161000
201903070064	Female	53	7/3/2019	0.58	115.8	1	4.1	280000
201909190131	Male	31	10/10/2019	12.38	5.1	5	4.2	205000
201809070036	Male	33	6/3/2019	8.68	7.6	5	4.2	190000
201707170228	Male	56	21-03-2018	6.19	10	5	4.2	303000
200806110173	Female	56	21-06-2018	4.21	11.6	5	4.2	174000
201807180004	Male	49	20-11-2018	5.15	12.8	5	4.2	199000
201804180051	Female	23	16-05-2018	0.55	145.8	1	4.2	240000
201901090058	Male	66	30-01-2019	7.87	7.4	5	4.3	258000

201903140019	Female	42	14-03-2019	4.96	10.2	5	4.3	189000
201910020070	Male	73	2/10/2019	1.4	52.9	3	4.3	235000
201904290113	Female	51	29-04-2019	0.75	86.7	2	4.3	337000
200507060068	Female	48	5/8/2019	0.72	92	1	4.3	249000
201305140007	Male	64	30-10-2018	0.87	94	1	4.3	278000
201911140138	Male	48	15-11-2019	0.84	103.8	1	4.3	231000
201605240023	Male	34	29-04-2019	0.83	112.9	1	4.3	261000
201901110010	Female	56	11/1/2019	0.56	119.2	1	4.3	378000
201801170121	Female	37	18-01-2018	0.55	132.4	1	4.3	308000
201811030017	Male	47	7/11/2018	0.51	185.5	1	4.3	525000
201901090138	Male	58	9/1/2019	6.97	8.7	5	4.4	257000
201612190086	Female	59	10/8/2018	4.83	9.8	5	4.4	195000
201807100007	Male	56	19-07-2018	5.72	11	5	4.4	275000
201809250020	Male	56	25-09-2018	5.01	12.8	5	4.4	246000
200505110075	Female	53	12/6/2018	2.35	23	4	4.4	259000
201203080074	Male	66	10/4/2019	2.7	25.3	4	4.4	223000
KGH-201503050099	Female	36	26-01-2019	0.47	159.6	1	4.4	105000
201807090028	Male	38	9/7/2018	6.92	9.6	5	4.5	120000
201905080039	Male	61	8/5/2019	4.67	13.7	5	4.5	120000
KGH-201501290079	Female	71	16-08-2018	1.63	33.1	3	4.5	246000
201603190033	Female	59	15-12-2018	0.51	131.4	1	4.5	357000
201812170039	Male	22	17-12-2018	0.77	134.5	1	4.5	268000
201807290032	Male	34	31-08-2018	2.86	27.1	4	4.6	282000
201811270023	Male	63	27-11-2018	1.29	59.9	3	4.6	244000
201904300025	Male	30	25-05-2019	9.79	6.7	5	4.7	157000
201808140022	Male	57	21-11-2019	7.14	8.5	5	4.7	216000
KGH-201503020012	Male	27	5/12/2018	8.1	8.5	5	4.7	220000
201907290007	Male	32	21-10-2019	7.73	8.7	5	4.7	167000
201810260080	Male	56	26-10-2018	5.49	11.5	5	4.7	360000
201810120078	Male	30	3/12/2018	5.81	12.3	5	4.7	337000
201911120059	Male	53	12/11/2019	3.57	19.2	4	4.7	419000
201907200038	Male	63	20-07-2019	3.13	21.5	4	4.7	337000
200003160036	Male	85	3/1/2019	1.52	46.6	3	4.7	167000
201806210002	Female	40	21-06-2018	0.45	164.3	1	4.7	279000
201806290051	Male	36	18-06-2019	10.39	6	5	4.8	160000
KGH-201512290069	Female	49	21-08-2018	6.33	7.5	5	4.8	219000
201801170072	Male	34	17-01-2018	7.51	8.9	5	4.8	538000
201903260042	Male	36	9/7/2019	6.55	10.3	5	4.8	249000
200112250116	Male	36	9/3/2018	5.64	12.2	5	4.8	223000
201904080052	Female	54	8/4/2019	2.18	25	4	4.8	305000
201305130058	Male	48	5/5/2018	1.24	66.2	2	4.8	201000

201305130058	Male	48	5/5/2018	1.23	66.9	2	4.8	201000
201803270094	Female	55	28-03-2018	0.86	72.9	2	4.8	373000
201706130058	Male	67	12/4/2018	0.75	110.6	1	4.8	166000
201609140092	Female	66	2/5/2018	0.38	180.4	1	4.8	353000
201812240052	Male	54	24-12-2018	7.98	7.5	5	4.9	294000
KGH-201504150058	Male	59	16-01-2018	7.51	7.9	5	4.9	180000
201704240141	Male	38	15-11-2019	7.79	8.3	5	4.9	328000
201806290051	Male	36	29-06-2018	7.71	8.5	5	4.9	185000
201903300054	Female	24	10/4/2019	6.3	8.7	5	4.9	246000
201808300015	Male	52	30-08-2018	6.08	10.4	5	4.9	282000
201807190004	Male	67	19-07-2018	3.65	17.8	4	4.9	251000
KGH-201504110120	Female	47	6/3/2018	0.98	64.8	2	4.9	340000
201806040130	Male	82	4/6/2018	1.06	71.2	2	4.9	247000
201805110028	Female	40	11/5/2018	0.53	136	1	4.9	273000
201606270082	Male	39	2/3/2019	12.19	4.9	5	5	242000
201806090089	Male	33	12/9/2018	9.14	7.1	5	5	187000
201903260079	Female	26	18-07-2019	7.24	7.3	5	5	502000
201812100100	Female	67	10/12/2018	5.82	7.7	5	5	169000
201808020013	Male	33	2/8/2018	8.09	8.2	5	5	321000
201810240067	Male	44	24-10-2018	5.66	11.7	5	5	228000
201805140011	Male	62	14-05-2018	5.24	11.9	5	5	324000
201410190048	Female	68	1/7/2019	3	16.5	4	5	293000
201906110020	Male	63	11/6/2019	2.71	25.4	4	5	223000
KGH-201506100030	Male	53	1/4/2019	2.31	31.7	3	5	191000
201811210111	Male	53	28-11-2018	1.91	39.4	3	5	289000
201912270070	Male	58	27-12-2019	1.22	64.9	2	5	238000
201901230042	Female	54	29-05-2019	0.69	94.4	1	5	282000
201905220041	Male	31	28-05-2019	9.52	6.9	5	5.1	229000
201911050078	Female	55	5/11/2019	3.82	13.1	5	5.1	447000
201904030041	Male	60	3/4/2019	4.17	15.6	4	5.1	221000
201012110053	Female	67	15-03-2019	0.96	61.7	2	5.1	354000
201002060102	Male	51	5/7/2018	1.16	70.7	2	5.1	298000
KGH-201503060017	Male	63	24-12-2019	1.04	76.8	2	5.1	183000
201009300035	Male	51	9/1/2018	0.98	85.8	2	5.1	239000
201806290047	Male	68	5/10/2018	0.78	105.4	1	5.1	339000
201910020100	Male	46	19-11-2019	0.71	127.2	1	5.1	220000
201911110111	Male	22	11/11/2019	0.8	128.7	1	5.1	210000
201806110006	Male	29	11/6/2018	12.32	5.2	5	5.2	162000
201911180004	Male	53	29-11-2019	8.67	6.9	5	5.2	170000
201807060050	Male	31	19-09-2018	8.55	7.8	5	5.2	115000
201912060088	Male	46	6/12/2019	5.11	13	5	5.2	283000

201907020065	Male	56	2/7/2019	3.23	21.3	4	5.2	302000
201802060058	Male	63	6/2/2018	1.62	46	3	5.2	257000
201904060047	Male	63	6/4/2019	1.37	55.9	3	5.2	226000
201903140048	Male	35	14-03-2019	1.22	72	2	5.2	301000
201910140051	Female	42	14-10-2019	0.67	102.8	1	5.2	178000
201806010083	Male	28	3/6/2018	0.71	140.6	1	5.2	308000
201605210027	Female	46	4/1/2018	8.58	5.3	5	5.3	181000
201802090039	Male	56	9/2/2018	9.75	5.9	5	5.3	178000
201805220100	Female	31	18-06-2019	0.99	69.6	2	5.3	257000
201908300036	Female	22	30-08-2019	0.88	85.5	2	5.3	354000
201802060095	Female	58	10/4/2019	9.5	4.5	5	5.4	345000
201812290076	Male	33	7/8/2019	10.9	5.8	5	5.4	156000
201011250056	Female	72	27-06-2018	7.2	5.9	5	5.4	294000
201704100054	Female	30	16-05-2018	7.76	6.5	5	5.4	204000
201612190031	Male	63	1/7/2019	8.57	6.7	5	5.4	227000
201605020041	Female	59	21-04-2018	6.35	7.2	5	5.4	223000
201805070053	Male	62	7/5/2018	6.79	8.8	5	5.4	335000
201607010057	Male	27	15-08-2018	6.13	11.8	5	5.4	273000
201911220024	Female	67	22-11-2019	3.54	13.7	5	5.4	262000
201111280184	Female	57	27-11-2018	2.98	17.3	4	5.4	221000
200711200031	Male	36	8/11/2018	1.26	68.9	2	5.4	300000
201703040106	Male	53	7/11/2018	1.01	82.3	2	5.4	406000
201808170022	Female	50	17-08-2018	0.71	92.8	1	5.4	372000
201806010015	Male	60	1/6/2018	0.89	92.8	1	5.4	288000
201903130004	Male	29	13-03-2019	13.49	4.7	5	5.5	186000
201902250007	Male	30	14-03-2019	10.4	6.3	5	5.5	160000
201707170228	Male	57	22-11-2018	9.03	6.5	5	5.5	265000
200704090068	Male	34	19-11-2018	8.69	7.5	5	5.5	195000
200806130074	Male	45	17-09-2018	5.5	12	5	5.5	202000
201912280061	Male	45	28-12-2019	5.39	12.3	5	5.5	181000
201809200037	Male	48	20-09-2018	4.47	15.1	4	5.5	221000
201803200098	Male	36	20-03-2018	4.53	15.7	4	5.5	207000
201806260060	Female	62	26-06-2018	2.18	24.3	4	5.5	360000
201609090044	Male	87	14-04-2018	1.87	36.5	3	5.5	184000
201805130024	Male	67	18-09-2019	1.5	49.7	3	5.5	306000
201405260003	Male	49	11/10/2019	1.41	56.9	3	5.5	202000
201802230041	Female	75	23-02-2018	0.65	94.6	1	5.5	366000
201805100013	Male	78	10/5/2018	0.83	95.4	1	5.5	188000
201801220097	Male	50	2/4/2018	8.34	7.3	5	5.6	221000
201804250039	Female	55	25-04-2018	5.62	8.4	5	5.6	341000
201812260120	Male	69	26-12-2018	2.03	34.8	3	5.6	268000
201903150090	Male	47	15-03-2019	2.1	36.2	3	5.6	165000
201903270065	Male	45	27-03-2019	1.66	47.9	3	5.6	360000
201906210079	Male	81	27-06-2019	1.35	54	3	5.6	361000
201808180003	Male	63	27-08-2018	1.2	65.1	2	5.6	413000

KGH-201509180054	Female	51	30-05-2018	0.92	68.5	2	5.6	270000
201807060044	Male	43	3/1/2019	1.18	71.7	2	5.6	301000
201009300035	Male	52	17-01-2019	0.97	86.5	2	5.6	221000
201605020127	Male	34	13-10-2018	0.71	135.2	1	5.6	419000
201804050025	Male	45	5/4/2018	0.63	146.6	1	5.6	229000
201906070090	Male	41	19-11-2019	7.65	8.4	5	5.7	235000
201904150229	Male	46	13-12-2019	7.45	8.4	5	5.7	170000
201812100005	Male	65	10/12/2018	3.95	16.4	4	5.7	256000
201804030063	Male	65	17-04-2018	3.72	17.5	4	5.7	216000
201012100062	Male	67	19-02-2019	2.42	28.6	4	5.7	256000
201801300072	Male	39	30-01-2018	1.02	86.6	2	5.7	260000
201703310074	Female	43	30-03-2019	0.61	114	1	5.7	570000
201810050023	Male	42	5/10/2018	0.66	140.9	1	5.7	208000
201704090016	Male	61	10/5/2019	8.54	6.8	5	5.8	224000
201803010010	Male	44	1/3/2018	8.68	7.1	5	5.8	231000
201812120018	Male	30	19-12-2018	8.37	8	5	5.8	238000
201804030028	Male	56	3/4/2018	2.34	30.9	3	5.8	255000
201811290039	Female	44	29-11-2018	1.84	31.7	3	5.8	288000
201012100062	Male	66	10/9/2018	2.19	32.2	3	5.8	194000
200712270071	Male	86	11/10/2019	1.51	46.9	3	5.8	184000
201307040041	Female	34	3/4/2018	0.63	115.2	1	5.8	352000
200504190008	Male	36	25-08-2018	0.67	142.9	1	5.8	215000
201905100074	Female	40	10/5/2019	8.3	5.7	5	5.9	243000
201807200016	Male	39	6/8/2018	9.75	6.4	5	5.9	409000
201805170069	Male	40	1/6/2018	8.56	7.4	5	5.9	215000
201912050042	Male	40	12/12/2019	8.15	7.8	5	5.9	223000
201911110009	Male	50	11/11/2019	4.72	14	5	5.9	370000
201909100025	Male	54	10/9/2019	2.65	26.9	4	5.9	209000
201801090119	Female	71	9/1/2018	1.88	28.1	4	5.9	396000
201911020044	Male	50	6/11/2019	1.53	51.5	3	5.9	400000
201901240030	Female	50	24-01-2019	1.11	55.4	3	5.9	375000
201905300043	Male	42	30-05-2019	1.46	56.4	3	5.9	256000
201801230060	Male	71	23-01-2018	1.17	65.4	2	5.9	339000
201709130030	Male	64	12/10/2019	0.87	94	1	5.9	190000
201905080033	Male	50	8/5/2019	0.76	115.6	1	5.9	254000
201706040014	Male	39	31-12-2018	8.54	7.5	5	6	260000
201809050083	Female	40	5/9/2018	6.53	7.5	5	6	416000
201909040007	Female	29	4/9/2019	3.38	17.1	4	6	216000
201203080074	Male	65	7/2/2019	2.68	25.6	4	6	197000
201809210071	Male	62	21-09-2018	1.57	47.9	3	6	356000
200905190079	Male	63	27-12-2018	1.46	51.9	3	6	357000
201909130052	Male	55	13-09-2019	1.25	63.8	2	6	306000
201807230095	Male	53	6/3/2019	1.21	66.8	2	6	284000
201805040061	Male	25	7/5/2019	1.24	75.6	2	6	199000

201007120027	Male	49	19-06-2018	1.1	75.7	2	6	277000
201706160051	Female	61	5/2/2019	0.78	79.9	2	6	269000
201906040013	Male	43	18-06-2019	10.52	5.7	5	6.1	135000
201801300043	Male	45	9/2/2018	8.72	7.1	5	6.1	181000
201809010072	Male	53	1/9/2018	8.33	7.2	5	6.1	223000
201903280109	Male	69	28-03-2019	7.91	7.3	5	6.1	372000
201807180004	Male	49	18-07-2018	7.61	8.1	5	6.1	247000
201911110011	Male	67	11/11/2019	4.32	14.7	5	6.1	454000
201811070080	Male	54	17-12-2018	4.17	16	4	6.1	182000
201907250093	Male	63	25-07-2019	2.75	25	4	6.1	366000
201904200002	Male	49	20-04-2019	2.62	27.8	4	6.1	426000
201912120061	Male	60	12/12/2019	2.33	30.6	3	6.1	220000
201807120060	Male	65	12/7/2018	1.53	48.9	3	6.1	387000
201910120074	Male	70	12/10/2019	1.22	62.5	2	6.1	204000
201205260069	Female	83	6/11/2019	0.72	82.4	2	6.1	257000
201805080079	Female	66	8/5/2018	7.8	5.5	5	6.2	287000
201903050080	Female	23	10/7/2019	8.61	6.1	5	6.2	189000
201612030027	Male	35	6/8/2018	5.67	12.2	5	6.2	160000
201807050046	Female	49	5/7/2018	3.87	13.2	5	6.2	331000
201902150006	Female	54	15-02-2019	2.16	25.3	4	6.2	308000
201810150068	Male	75	16-10-2018	2.13	32.4	3	6.2	248000
201801250037	Male	23	25-01-2018	2.12	41.4	3	6.2	209000
201909190009	Male	63	19-09-2019	1.42	53.6	3	6.2	227000
201904230064	Male	60	30-05-2019	1.35	57.4	3	6.2	321000
201704080116	Male	54	6/12/2018	1.08	75.9	2	6.2	472000
201806010074	Male	34	2/4/2019	14.46	4.2	5	6.3	290000
201801220092	Male	47	23-01-2018	9.05	6.7	5	6.3	375000
201802080022	Male	33	8/2/2018	8.98	7.3	5	6.3	317000
201810010167	Male	61	1/10/2018	7.6	7.8	5	6.3	216000
201704040046	Female	43	24-07-2019	5.79	8.5	5	6.3	287000
201905140024	Male	39	14-06-2019	6.53	10.2	5	6.3	285000
201106300140	Male	63	8/8/2019	3.28	20.4	4	6.3	192000
201910290016	Male	70	29-10-2019	2.91	22.9	4	6.3	236000
201902080014	Female	64	8/2/2019	2.09	25.4	4	6.3	326000
201807180073	Male	66	18-07-2018	2.61	26.3	4	6.3	366000
201005270073	Male	41	6/12/2019	2.77	27.1	4	6.3	299000
200611300023	Male	55	4/3/2019	1.64	46.7	3	6.3	207000
201712230114	Female	49	11/12/2018	1.11	55.6	3	6.3	194000
KGH-201506170085	Male	58	7/3/2019	1.31	59.8	3	6.3	204000
201810230056	Male	69	23-10-2018	1.24	61.5	2	6.3	254000
201806260058	Male	60	26-06-2018	1.16	68.4	2	6.3	281000
201802270048	Female	52	27-02-2018	0.85	74.8	2	6.3	216000
201901040069	Male	65	4/1/2019	0.87	93.8	1	6.3	291000
201611110107	Male	18	17-02-2018	0.84	126.7	1	6.3	213000

201804130043	Male	37	16-05-2018	12.26	5	5	6.4	223000
201805220078	Female	65	13-08-2018	5.93	7.6	5	6.4	222000
201808010004	Male	63	1/8/2018	6.39	9.4	5	6.4	153000
201811230015	Male	44	23-11-2018	5.42	12.3	5	6.4	257000
201806270072	Male	61	5/11/2019	2.96	23.1	4	6.4	333000
201803280058	Male	70	29-03-2018	2.78	24.2	4	6.4	234000
201806290054	Female	45	29-06-2018	1.75	33.5	3	6.4	262000
201806270072	Male	60	27-06-2018	1.87	39.4	3	6.4	275000
201803030018	Male	60	3/3/2018	1.86	39.6	3	6.4	299000
201008090116	Male	66	3/6/2019	0.83	98.7	1	6.4	235000
201811260079	Male	65	26-11-2018	9.71	5.8	5	6.5	254000
201308050036	Female	66	20-04-2018	3.87	12.4	5	6.5	181000
201910300094	Male	82	30-10-2019	1.18	62.9	2	6.5	382000
201309010040	Female	39	9/5/2019	0.75	91.6	1	6.5	287000
201801190014	Male	40	19-01-2018	0.81	112.4	1	6.5	183000
KGH-201503020141	Male	61	16-11-2018	0.72	118.1	1	6.5	407000
201805090005	Male	29	9/5/2018	0.82	118.3	1	6.5	210000
201809100018	Female	45	10/9/2018	0.58	119.7	1	6.5	407000
201906100028	Female	67	10/6/2019	10.11	4.1	5	6.6	272000
201912190031	Female	43	31-12-2019	8	5.8	5	6.6	224000
201810080032	Male	60	8/10/2018	7.98	7.4	5	6.6	182000
201805050061	Male	55	30-07-2018	7.35	8.3	5	6.6	288000
201905020046	Male	47	4/5/2019	6.83	9.3	5	6.6	242000
201901120106	Male	49	15-01-2019	5.5	11.8	5	6.6	163000
201910110045	Male	65	11/10/2019	4.04	15.9	4	6.6	225000
201407080002	Male	62	14-11-2018	2.4	29.3	4	6.6	200000
201709140003	Male	59	3/1/2018	1.61	47	3	6.6	173000
201912120007	Male	75	12/12/2019	1.41	52.2	3	6.6	185000
201007120115	Male	72	29-12-2018	1.2	63.4	2	6.6	249000
201912050041	Male	32	6/12/2019	1.04	88.1	2	6.6	238000
201803080050	Male	52	17-05-2019	0.84	102.2	1	6.6	339000
201910140071	Male	30	14-10-2019	0.75	130.2	1	6.6	291000
201808140039	Male	34	14-08-2018	11.86	5.2	5	6.7	192000
201903250006	Male	18	24-06-2019	11.12	6.4	5	6.7	171000
201705260025	Male	63	8/2/2019	8.79	6.5	5	6.7	245000
201903260079	Female	26	26-03-2019	6.03	9	5	6.7	226000
201807230050	Female	58	23-07-2018	4.57	10.5	5	6.7	563000
201809140046	Male	83	14-09-2018	1.54	46.2	3	6.7	276000
201711270026	Male	66	4/11/2019	0.78	106	1	6.7	206000
201911180175	Female	24	19-11-2019	0.7	109.4	1	6.7	290000
201802270007	Male	42	27-02-2018	9.82	6.2	5	6.8	328000
201808290074	Male	42	28-05-2019	8.49	7.4	5	6.8	165000
201802090018	Male	47	9/2/2018	8.21	7.5	5	6.8	212000
201806070119	Male	45	7/6/2018	7.79	8	5	6.8	210000

201903150006	Male	35	15-03-2019	7.95	8.3	5	6.8	172000
201811100044	Female	28	10/11/2018	2.11	29.7	4	6.8	327000
201902250079	Male	77	25-02-2019	2.07	33.3	3	6.8	256000
201909160003	Male	44	16-09-2019	2.08	37.1	3	6.8	247000
201801050096	Male	37	25-06-2018	1.64	50.6	3	6.8	216000
200104300002	Male	62	20-09-2018	1.35	57	3	6.8	191000
201912310076	Female	71	31-12-2019	1.01	57.5	3	6.8	301000
201805020021	Male	42	2/5/2018	1.23	68.7	2	6.8	210000
201909070062	Male	46	7/9/2019	1.21	68.7	2	6.8	339000
201710050132	Male	35	30-07-2018	1	90.5	1	6.8	249000
201911120051	Male	54	12/11/2019	10.41	5.6	5	6.9	217000
201802170048	Male	40	17-02-2018	8.94	7	5	6.9	251000
201902180046	Female	32	18-02-2019	5.47	9.6	5	6.9	252000
201906120003	Male	48	12/6/2019	2.41	30.8	3	6.9	289000
201409020093	Male	58	23-03-2018	2.24	32.2	3	6.9	226000
201901030050	Female	47	3/1/2019	1.37	44	3	6.9	360000
201909200068	Female	60	20-09-2019	1.13	52.3	3	6.9	294000
201002170013	Male	28	13-02-2019	0.65	155.7	1	6.9	424000
201802150034	Male	25	15-02-2018	16.45	3.8	5	7	190000
201911280037	Male	34	16-12-2019	12.98	4.7	5	7	181000
201903160004	Female	31	16-03-2019	8.54	5.8	5	7	168000
201907250057	Male	48	25-07-2019	7.62	8.1	5	7	164000
201812200110	Male	55	20-12-2018	5.62	11.3	5	7	105000
201902070010	Male	22	1/3/2019	6.31	11.9	5	7	290000
201810110054	Male	41	11/10/2018	1.59	51.3	3	7	285000
201006140015	Male	37	5/11/2019	0.78	119.2	1	7	231000
201412110113	Male	31	11/6/2018	8.4	8	5	7.1	210000
201803060058	Male	48	6/3/2018	7.21	8.7	5	7.1	475000
201405260003	Male	50	19-06-2018	6.78	9.2	5	7.1	294000
201909270084	Female	47	27-09-2019	3.2	16.5	4	7.1	340000
201701130051	Male	43	16-08-2018	3.26	22.2	4	7.1	278000
201801020094	Male	59	2/1/2018	2.51	28.2	4	7.1	222000
201812050003	Male	43	5/12/2018	2.6	28.8	4	7.1	165000
201807020026	Female	60	2/7/2018	1.66	33.5	3	7.1	342000
201603010093	Male	47	28-02-2019	1.56	51	3	7.1	418000
KGH-201501260091	Male	69	28-09-2019	1.31	57.8	3	7.1	294000
201906080046	Male	43	8/6/2019	1.19	71	2	7.1	345000
201907180002	Male	22	30-07-2019	16.57	3.9	5	7.2	211000
201404280118	Male	42	5/7/2018	11.95	5	5	7.2	194000
201906170073	Male	74	17-06-2019	7.38	7.7	5	7.2	191000
201901080019	Male	55	8/1/2019	2.94	23.8	4	7.2	277000
201912020079	Female	69	3/12/2019	1.78	30.1	3	7.2	337000
201306160063	Male	60	21-03-2018	1.49	51.2	3	7.2	155000
201903120035	Male	65	12/3/2019	1.19	65.3	2	7.2	256000

201903180135	Male	57	18-03-2019	1.2	66.4	2	7.2	183000
201810290122	Male	49	29-10-2018	1.01	83.6	2	7.2	231000
201806290091	Male	57	29-06-2018	0.93	89.2	2	7.2	250000
201809030045	Male	57	3/9/2018	0.93	89.2	2	7.2	292000
201904160041	Male	30	16-04-2019	0.87	109.7	1	7.2	329000
201810300030	Female	36	30-10-2018	9.8	4.8	5	7.3	120000
201809040031	Male	35	4/9/2018	12.01	5.1	5	7.3	170000
201902250137	Male	42	7/3/2019	10.28	5.9	5	7.3	356000
201811210053	Male	48	21-11-2018	5.05	13.1	5	7.3	382000
201904260097	Male	50	26-04-2019	3.22	21.8	4	7.3	222000
201806120047	Male	38	12/6/2018	0.8	115.2	1	7.3	325000
201907250121	Male	49	25-07-2019	12.72	4.5	5	7.4	329000
201805010045	Male	48	28-05-2018	10.04	5.9	5	7.4	254000
201905090004	Male	56	9/5/2019	9.37	6.2	5	7.4	425000
201801090081	Male	75	9/1/2018	4.04	15.5	4	7.4	237000
201804120075	Male	64	12/4/2018	3.79	17.2	4	7.4	210000
201910230002	Male	56	23-10-2019	2.83	24.8	4	7.4	196000
201805050019	Male	55	5/5/2018	2.05	36.1	3	7.4	208000
201810190080	Female	24	19-10-2018	1.47	46.5	3	7.4	228000
201811130038	Male	60	13-11-2018	1.59	47.5	3	7.4	285000
201808270049	Male	48	27-08-2018	1.45	55.3	3	7.4	180000
201912270048	Male	76	27-12-2019	1.15	65.8	2	7.4	265000
201002200132	Male	63	13-12-2019	1.16	67.7	2	7.4	178000
201807090015	Male	36	9/7/2018	1	90	2	7.4	257000
201806250006	Male	52	25-06-2018	10.6	5.5	5	7.5	276000
201805110054	Male	34	12/5/2018	8.1	8.1	5	7.5	186000
201801030011	Female	54	3/1/2018	4.66	10.4	5	7.5	379000
201912050046	Male	34	5/12/2019	3.09	24.8	4	7.5	295000
201612200122	Male	62	7/3/2019	1.96	37.1	3	7.5	152000
201908310048	Male	35	31-08-2019	1.14	77.8	2	7.5	266000
201805290035	Male	65	29-05-2018	0.77	107.9	1	7.5	256000
201902110062	Male	50	11/2/2019	0.61	149	1	7.5	246000
201806290051	Male	36	21-03-2019	22.18	2.5	5	7.6	150000
201808200065	Male	65	20-08-2018	9.19	6.2	5	7.6	428000
201912250012	Male	22	25-12-2019	1.85	48.9	3	7.6	202000
201308210048	Male	38	4/9/2018	1.5	55.8	3	7.6	368000
201907010190	Male	63	10/7/2019	1.25	62.1	2	7.6	465000
201906050039	Male	64	5/6/2019	0.94	86	2	7.6	191000
201207170037	Male	38	8/3/2018	0.89	101.8	1	7.6	251000
201803050032	Female	51	5/3/2018	4.45	11.1	5	7.7	209000
201702060005	Male	54	15-06-2018	2.8	25.3	4	7.7	159000
201805090072	Male	27	9/5/2018	3	26.9	4	7.7	193000
201610270041	Male	65	22-06-2018	1.38	55.1	3	7.7	294000
201804050046	Male	70	5/4/2018	9.89	5.6	5	7.8	220000
201810080084	Male	38	8/10/2018	9.79	6.4	5	7.8	226000

201802020030	Female	46	2/2/2018	6.03	8	5	7.8	332000
201802210042	Male	52	21-02-2018	5.59	11.5	5	7.8	75000
200503240007	Male	66	7/1/2019	0.94	85.5	2	7.8	244000
201803120087	Male	40	12/3/2018	37.26	1.4	5	7.9	155000
201704240141	Male	37	29-01-2019	10.87	5.7	5	7.9	375000
201812260077	Male	56	26-12-2018	7.87	7.6	5	7.9	181000
201908240043	Male	50	24-08-2019	3.46	20.1	4	7.9	260000
201803070005	Male	44	7/3/2018	3.5	20.4	4	7.9	273000
201907120041	Male	45	12/7/2019	12.3	4.8	5	8	165000
201902280022	Male	54	31-05-2019	9.42	6.2	5	8	182000
201903230155	Female	60	23-03-2019	4.36	11	5	8	342000
201805310095	Female	50	24-11-2018	2.33	23.5	4	8	192000
201810240091	Female	73	24-10-2018	2.05	25.3	4	8	372000
201810020030	Male	71	2/10/2018	1.4	53.2	3	8	151000
201903220095	Female	31	22-03-2019	6.47	8	5	8.1	160000
201704230016	Male	62	31-01-2018	6.25	9.7	5	8.1	187000
201608290069	Male	50	9/4/2018	5.02	13.1	5	8.1	215000
KGH-201501120046	Female	66	6/8/2018	2.43	21.2	4	8.1	171000
201909030098	Male	42	3/9/2019	3.3	22	4	8.1	309000
201901020039	Female	33	2/1/2019	2.32	25.7	4	8.1	191000
201809030117	Male	70	3/9/2018	2.59	26.2	4	8.1	278000
201806090023	Male	63	9/6/2018	1.73	42.7	3	8.1	176000
201909140132	Female	34	21-09-2019	1.25	52.2	3	8.1	299000
201901170098	Female	43	17-01-2019	0.75	89.8	2	8.1	263000
201812040047	Female	40	4/12/2018	12.24	3.6	5	8.2	75000
201904230028	Male	45	23-04-2019	9.44	6.4	5	8.2	187000
201810260042	Male	68	26-10-2018	6.61	8.9	5	8.2	152000
201108220073	Male	57	7/12/2018	6.6	9.3	5	8.2	205000
201802100114	Male	33	19-07-2018	6.43	10.7	5	8.2	303000
KGH-201412170097	Female	42	17-07-2019	1.77	33.5	3	8.2	262000
201803170007	Female	63	3/5/2018	0.61	105.5	1	8.2	405000
201806010074	Male	34	1/6/2018	12.34	5	5	8.3	258000
201904240030	Male	44	11/9/2019	9.78	6.2	5	8.3	191000
201906150015	Male	63	15-06-2019	8.01	7.3	5	8.3	271000
KGH-201412260009	Male	57	8/1/2019	7.56	7.9	5	8.3	260000
201904090099	Male	54	9/4/2019	4.52	14.5	5	8.3	321000
201212290052	Male	65	1/1/2018	3.39	19.5	4	8.3	271000
199905280088	Male	62	15-02-2018	2.52	27.7	4	8.3	268000
201411180136	Male	73	15-05-2018	2.22	31.1	3	8.3	421000
201910290020	Male	71	29-10-2019	1.69	42.8	3	8.3	194000
201809240047	Female	48	3/7/2019	1.17	52.6	3	8.3	267000
200812290221	Male	71	3/4/2018	1.08	71.8	2	8.3	256000

201805240125	Male	68	25-05-2018	0.98	81	2	8.3	237000
201911070096	Male	73	7/11/2019	11.05	4.9	5	8.4	210000
201807020002	Male	58	2/7/2018	5.28	12	5	8.4	412000
201804200046	Female	35	20-04-2018	2.7	21.3	4	8.4	420000
201812240120	Male	62	24-12-2018	3.09	21.9	4	8.4	225000
201903160004	Female	31	31-05-2019	11.29	4.2	5	8.5	194000
201907010012	Male	56	1/7/2019	4.47	14.6	5	8.5	194000
201909040069	Male	48	4/9/2019	2.76	26.3	4	8.5	342000
201803010118	Male	43	1/3/2018	2.37	32.1	3	8.5	303000
201811240051	Male	54	24-11-2018	1.79	42.3	3	8.5	390000
201904290018	Male	45	29-04-2019	24.39	2.2	5	8.6	203000
201808190012	Male	63	20-08-2018	5.69	10.8	5	8.6	262000
201812040053	Male	75	4/12/2018	5.26	11.4	5	8.6	374000
KGH-201511020042	Male	56	19-07-2019	4.12	16.1	4	8.6	167000
KGH-201506260060	Male	67	30-08-2018	3.97	16.2	4	8.6	264000
201808060058	Male	63	6/8/2018	2.3	30.7	3	8.6	253000
201202260058	Male	60	12/4/2019	2.2	32.7	3	8.6	233000
201006210015	Male	74	27-08-2019	0.81	99.2	1	8.6	302000
201410270031	Female	30	4/1/2018	9.66	5.1	5	8.7	167000
201809030075	Female	33	3/9/2018	6.8	7.4	5	8.7	280000
201802210041	Male	30	21-02-2018	4.5	16.5	4	8.7	252000
201910030153	Male	60	3/10/2019	3.43	19.6	4	8.7	395000
201811090088	Male	66	9/11/2018	1.7	43.1	3	8.7	150000
201809010052	Male	51	1/9/2018	1.69	45.8	3	8.7	192000
201808070101	Male	68	9/8/2018	1.59	46.3	3	8.7	280000
KGH-201505310059	Female	49	1/8/2018	1.3	46.3	3	8.7	150000
200606060109	Male	44	6/9/2018	1.25	66.8	2	8.7	208000
201911200111	Male	47	20-11-2019	19.06	2.8	5	8.8	204000
201906210006	Male	41	21-06-2019	4.65	14.9	5	8.8	209000
201205220022	Male	63	17-04-2019	1.32	58.3	3	8.8	308000
201907120041	Male	45	22-07-2019	13.51	4.3	5	8.9	172000
201803070076	Male	45	7/3/2018	11.38	5.2	5	8.9	393000
201806190023	Female	44	19-06-2018	2.8	19.5	4	8.9	205000
201902250007	Male	30	25-02-2019	18.06	3.3	5	9	227000
201903090015	Male	43	9/3/2019	14.19	4.1	5	9	255000
201810290103	Male	52	29-10-2018	1.09	75.6	2	9	269000
201801260154	Male	42	26-01-2018	21.77	2.5	5	9.2	125000
200609110076	Female	39	9/2/2019	8.2	5.8	5	9.2	140000
201801080107	Male	62	8/1/2018	8.48	6.8	5	9.2	157000
201906080048	Female	55	8/7/2019	6.36	7.2	5	9.2	159000
201801090002	Male	50	9/1/2018	4.06	16.7	4	9.2	386000
201208170115	Male	61	5/6/2018	3.78	17.4	4	9.2	320000

201907110017	Male	61	11/7/2019	2.99	22.8	4	9.2	235000
201909300168	Male	47	21-10-2019	1.43	56.4	3	9.2	217000
201905230071	Male	36	23-05-2019	0.96	94.4	1	9.2	357000
201808030012	Male	42	3/8/2018	26.15	2	5	9.3	202000
201809040032	Male	48	4/9/2018	10.06	5.9	5	9.3	231000
201911110020	Male	54	11/11/2019	7.15	8.6	5	9.4	368000
201911260058	Male	36	26-11-2019	1.27	68.3	2	9.4	188000
201901240009	Male	38	24-01-2019	12.85	4.7	5	9.5	208000
201908270047	Male	27	27-08-2019	10.6	6.3	5	9.5	247000
201802220028	Male	60	22-02-2018	6.91	8.7	5	9.5	193000
201908050018	Male	29	5/8/2019	4.93	14.9	5	9.5	153000
201808070123	Female	47	7/8/2018	17.68	2.3	5	9.6	352000
201603140121	Female	43	12/9/2018	16.19	2.6	5	9.6	238000
200908280037	Male	54	8/3/2019	11.77	4.8	5	9.6	438000
201804160056	Male	65	16-04-2018	10.49	5.3	5	9.6	304000
201806290018	Female	28	14-09-2018	6.59	8	5	9.6	359000
201902180104	Male	68	18-02-2019	5.15	11.9	5	9.6	262000
201809060046	Male	54	6/9/2018	1.68	45.6	3	9.6	198000
201801050041	Male	42	5/1/2018	1.19	71.4	2	9.7	254000
201901300036	Male	34	30-01-2019	10.26	6.2	5	9.8	215000
201303050012	Male	35	15-07-2019	8.96	7.2	5	9.9	219000
201804130021	Female	41	13-04-2018	4.7	10.9	5	9.9	297000
KGH-201506260060	Male	68	13-07-2019	5.28	11.6	5	9.9	374000
201706220110	Male	49	5/2/2018	5.28	12.4	5	9.9	120000
201902080043	Male	60	8/2/2019	1.26	62.1	2	9.9	218000
201307160068	Male	67	21-11-2018	10.77	5.1	5	10	172000
201005230020	Male	59	12/9/2019	10.53	5.4	5	10	243000
201905060102	Male	45	4/9/2019	8	7.8	5	10	297000
201904020070	Male	64	2/4/2019	2.37	29.6	4	10.1	353000
201211130010	Male	56	28-08-2019	2.02	36.6	3	10.1	230000
201809070158	Female	44	15-10-2018	13.72	3.1	5	10.2	282000
201810130118	Male	60	13-10-2018	7.15	8.4	5	10.2	177000
201907260076	Male	47	27-07-2019	2.06	37	3	10.2	293000
201909190043	Male	65	19-09-2019	2.23	31.6	3	10.3	160000
201812110042	Male	35	12/12/2018	18.63	3.1	5	10.5	237000
201803280096	Male	58	28-03-2018	7.98	7.4	5	10.5	483000
201907100002	Male	21	10/7/2019	18.4	3.5	5	10.6	456000
201911200060	Female	73	20-11-2019	2.7	18.4	4	10.6	331000
201604050153	Male	58	22-01-2019	3.06	22.5	4	10.6	341000
201807300118	Male	73	30-07-2018	7.46	7.7	5	10.7	340000
201812190054	Male	63	19-12-2018	2.94	23.1	4	10.7	224000
201803220006	Male	51	22-03-2018	2.42	30.2	3	10.7	320000
201411260038	Female	35	29-10-2018	13.43	3.4	5	10.8	201000
201804110036	Male	66	11/4/2018	9.34	6	5	10.8	165000

201805090039	Male	23	9/5/2018	5.18	14.8	5	10.8	276000
201105110165	Male	41	19-02-2019	3.9	18.2	4	10.8	262000
201804250038	Female	45	25-04-2018	4.06	12.7	5	10.9	294000
201903140041	Male	70	14-03-2019	2.51	27.2	4	10.9	275000
201802070113	Male	26	7/2/2018	21.45	2.8	5	11	190000
201705230088	Male	60	1/11/2019	8.37	7	5	11	310000
201808080021	Male	33	8/8/2018	7.61	8.8	5	11	492000
201712270034	Female	56	7/3/2018	1.47	39.1	3	11	245000
201805160051	Male	60	16-05-2018	7.24	8.3	5	11.1	155000
201803290055	Male	58	29-03-2018	1.94	38	3	11.2	503000
201910050146	Male	50	5/10/2019	17.65	3.1	5	11.3	292000
201704270021	Female	67	10/10/2019	3.74	12.8	5	11.4	293000
201912050022	Female	65	5/12/2019	1.81	29.9	4	11.4	175000
201902220047	Male	35	22-02-2019	1.58	53.4	3	11.4	80000
201812080079	Female	49	8/12/2018	3.83	13.3	5	11.5	352000
200205290053	Male	66	18-01-2019	2.19	32.2	3	11.6	206000
201907180002	Male	22	18-07-2019	11.53	5.9	5	12	225000
201807040048	Female	50	4/7/2018	4.74	10.4	5	12.2	325000
201603190028	Male	76	10/10/2019	3.42	18.7	4	12.2	390000
201901220045	Male	75	22-01-2019	6.36	9.2	5	12.3	205000
201903120070	Female	39	12/3/2019	8.4	5.6	5	12.5	284000
201010060059	Female	58	6/2/2019	3.07	16.6	4	12.6	158000
201906120102	Male	34	17-06-2019	1.75	47.7	3	12.8	254000
201803060035	Male	63	6/3/2018	0.65	132.1	1	12.9	200000
201911260052	Female	80	26-11-2019	4.22	10.8	5	14.1	252000
201909110055	Male	63	11/9/2019	8.62	6.7	5	14.3	520000
201806060058	Male	45	6/6/2018	23.18	2.3	5	14.9	156000
201806220067	Male	33	23-06-2018	4.5	16.1	4	15.1	477000
